# Supplementary material for: A thermally activated and highly miscible dopant for n-type organic thermoelectrics
Source: Nat Commun. 2020 Jul 3;11:3292. doi: 10.1038/s41467-020-17063-1 (PMC7335177; doi:10.1038/s41467-020-17063-1)
Supplement: Supplementary file 1 — Supplementary Information [file 41467_2020_17063_MOESM1_ESM.pdf]

Supplementary Information

A thermally activated and highly miscible dopant for n-type organic thermoelectrics

C.-Y. Yang et al.

Contents:

Supplementary Figures 1-67
Supplementary Tables 1-6
Supplementary Notes 1-11
Supplementary Methods
Supplementary References

Supplementary Figures

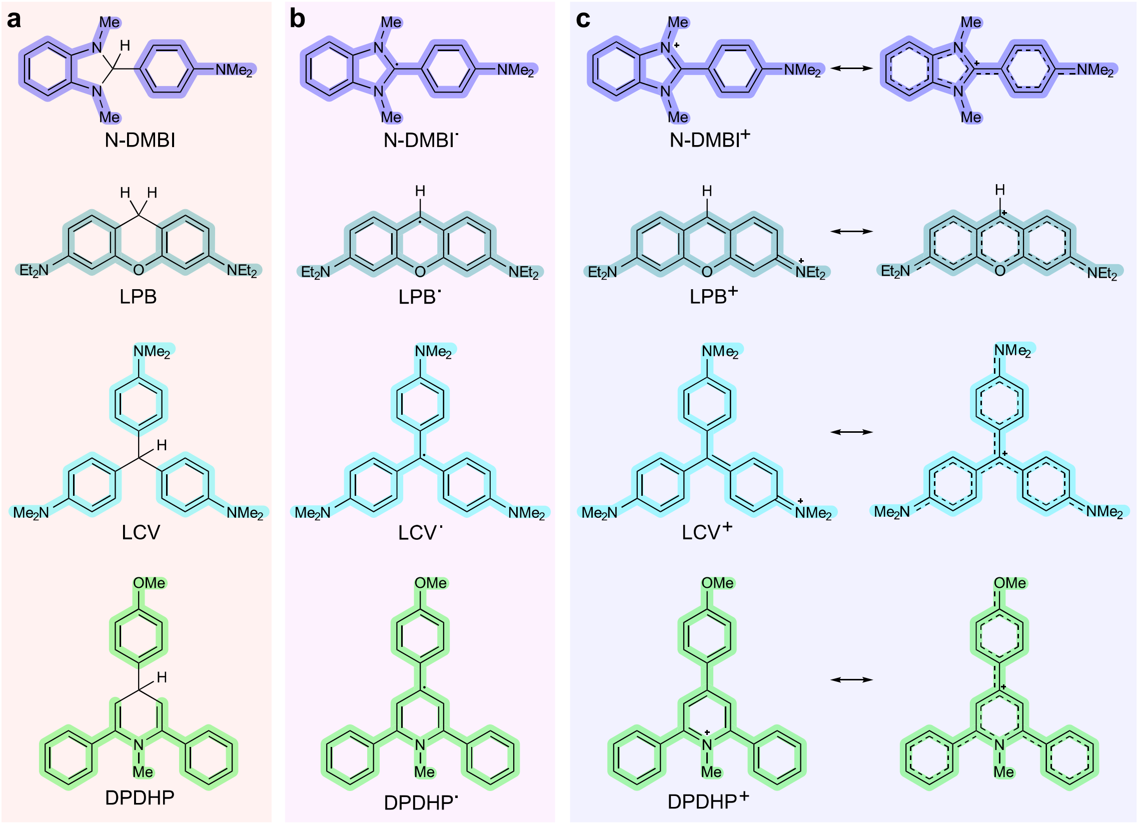

Supplementary Figure 1 | Hydride dopants. a-c Chemical structures of reported hydride dopants (**a**, including N-DMBI, LPB, LCV, and DPDHP), their corresponding radicals (**b**) and cations (**c**).

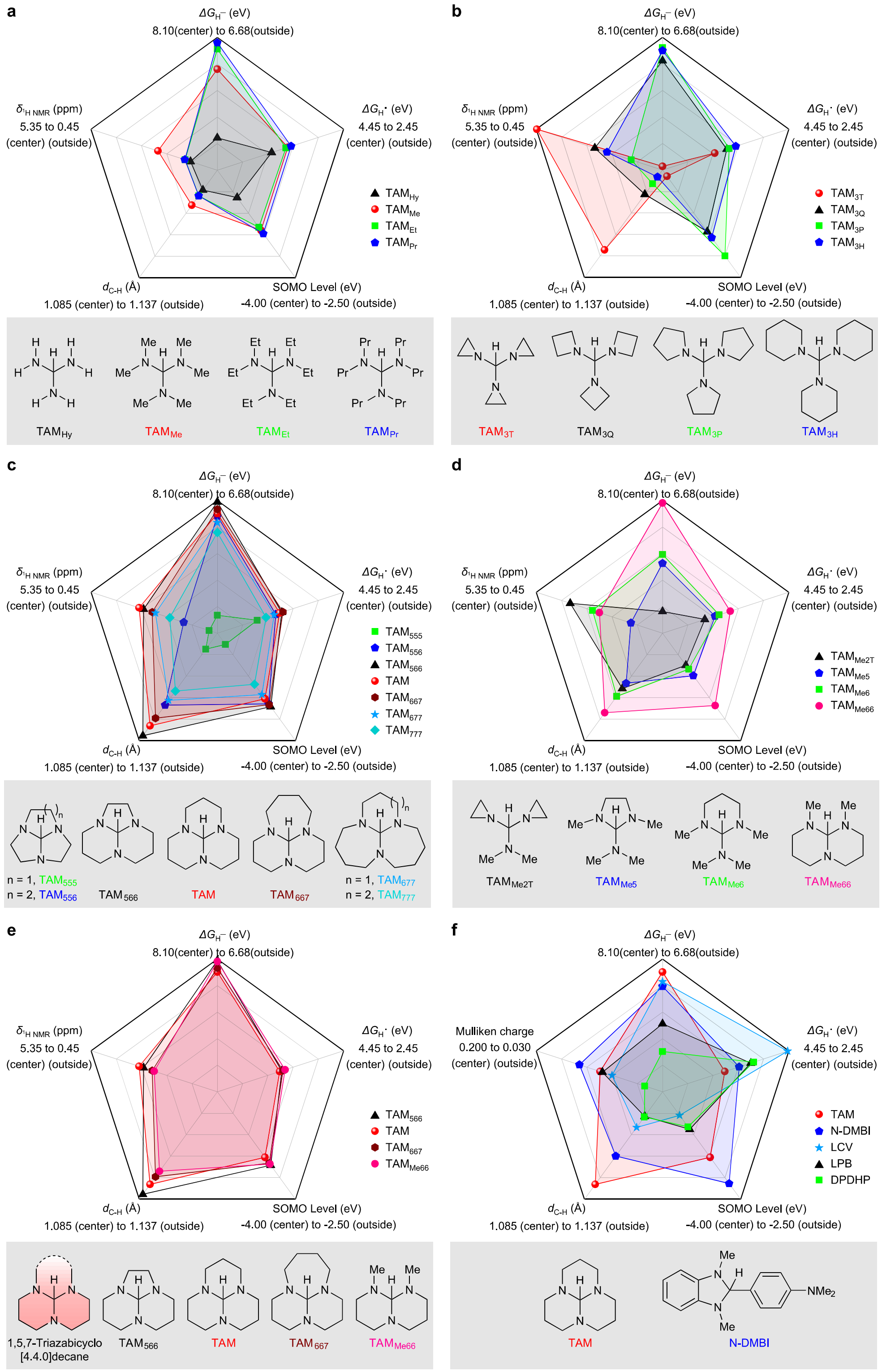

Supplementary Figure 2 | N-doping ability prediction. a-f Radar charms for predicting n-doping ability of TAMs (**a-e**) and reported hydride dopants (**f**). Alkyl substitutions on TAMs include hydrogen/linear alkyls (**a**), cyclic alkyls (**b**), fused ring alkyls (**c**), combinations of rings and methyl groups (**d**), and 1,5,7-triazabicyclo[4.4.0]decane derivatives (**e**). Evaluating indexes includes DFT calculated Gibbs free energy change in hydride-transfer half-reaction (ΔG_{H^-}) and hydrogen-atom-transfer half-reaction ($\Delta G_{H^\bullet}$), SOMO level of dopant radical, charge distribution (Mulliken charge or ^{1}H-NMR chemical shift) of reactive hydrogen, and hydrogen-carbon bond length ($d_{C\text{-}H}$).

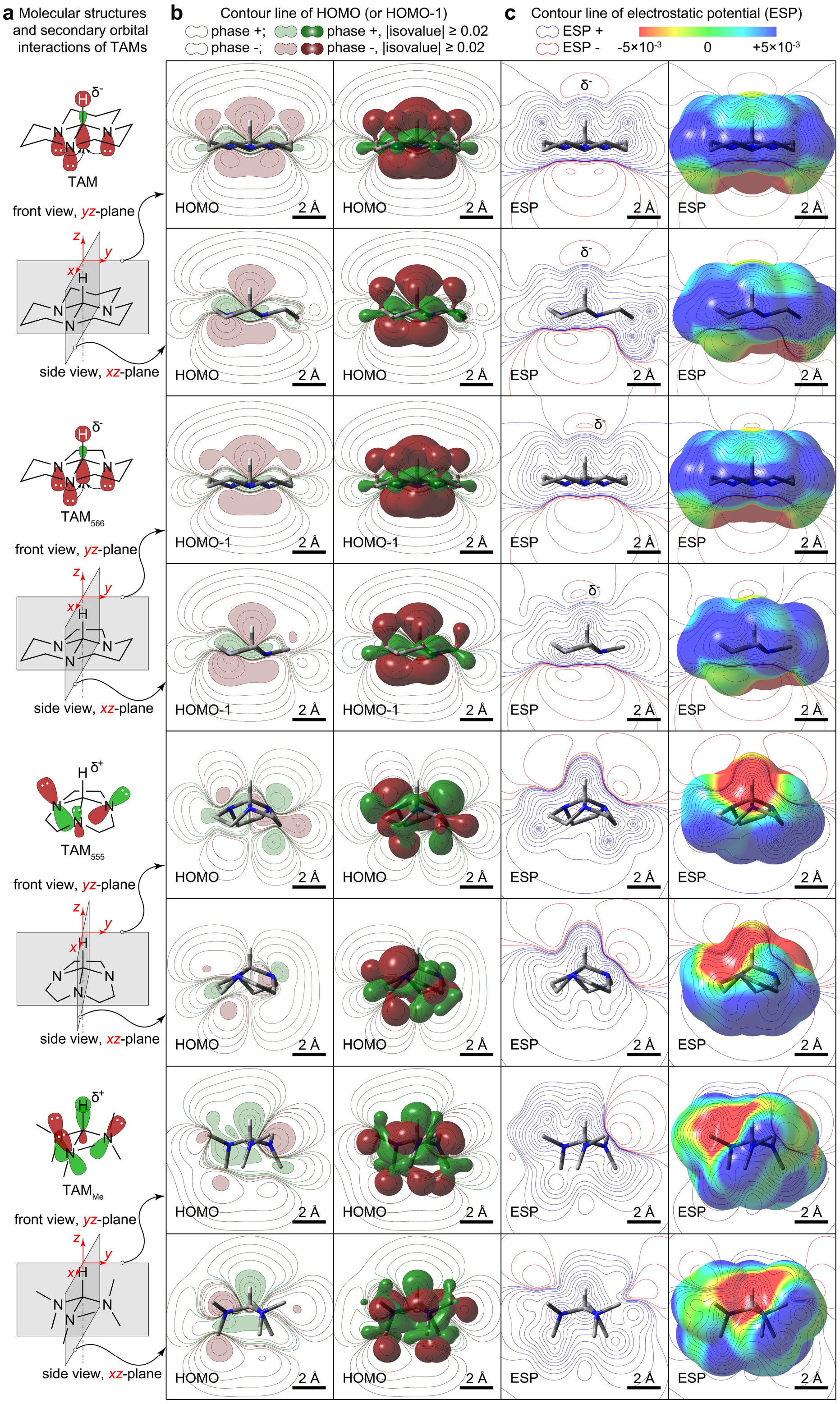

Supplementary Figure 3 | Secondary orbital interactions. **a** Stereoelectronic effect and secondary orbital interactions in TAM, TAM_{566}, TAM_{555} and TAM_{Me}. **b** Calculated HOMO and HOMO contour line. **c** Calculated electrostatic potential (ESP) and ESP contour line. The calculation performed under B3LYP/6-311+G(d,p) level. TAM_{566} and TAM have negative partial charges around the surface of their active hydrogen. These negative partial charges come from specific conformation induced secondary orbital interactions.

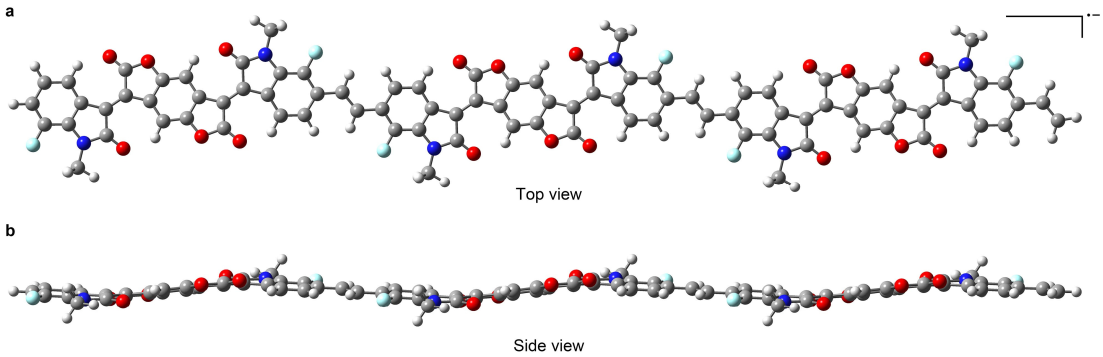

Supplementary Figure 4 | Polymer anion geometry. a-b DFT-optimized molecular structures of the FBDPPV trimer radical anion on top view (a) and side view (b). The calculation performed under B3LYP/6-31G(d), long alkyl chains were instead by methyl to simplify calculation. The optimized polymer anion structure are used in constructing of doped polymer super cell for molecular dynamics.

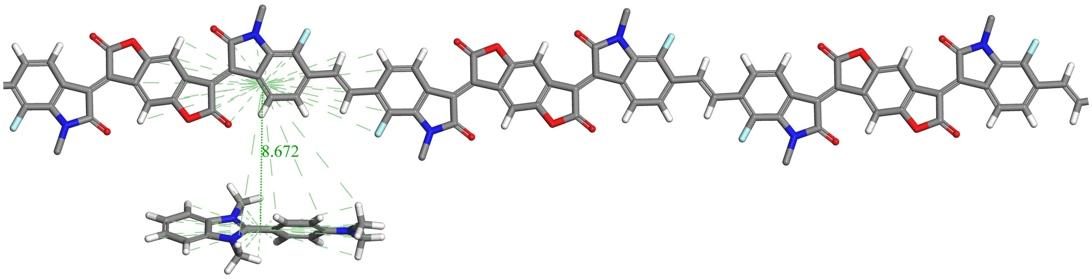

Supplementary Figure 5 | Statistics of dopant cation-backbone distances. Illustration of distance measuring method of dopant cation and its nearest polymer conjugated backbone (unit: Å).

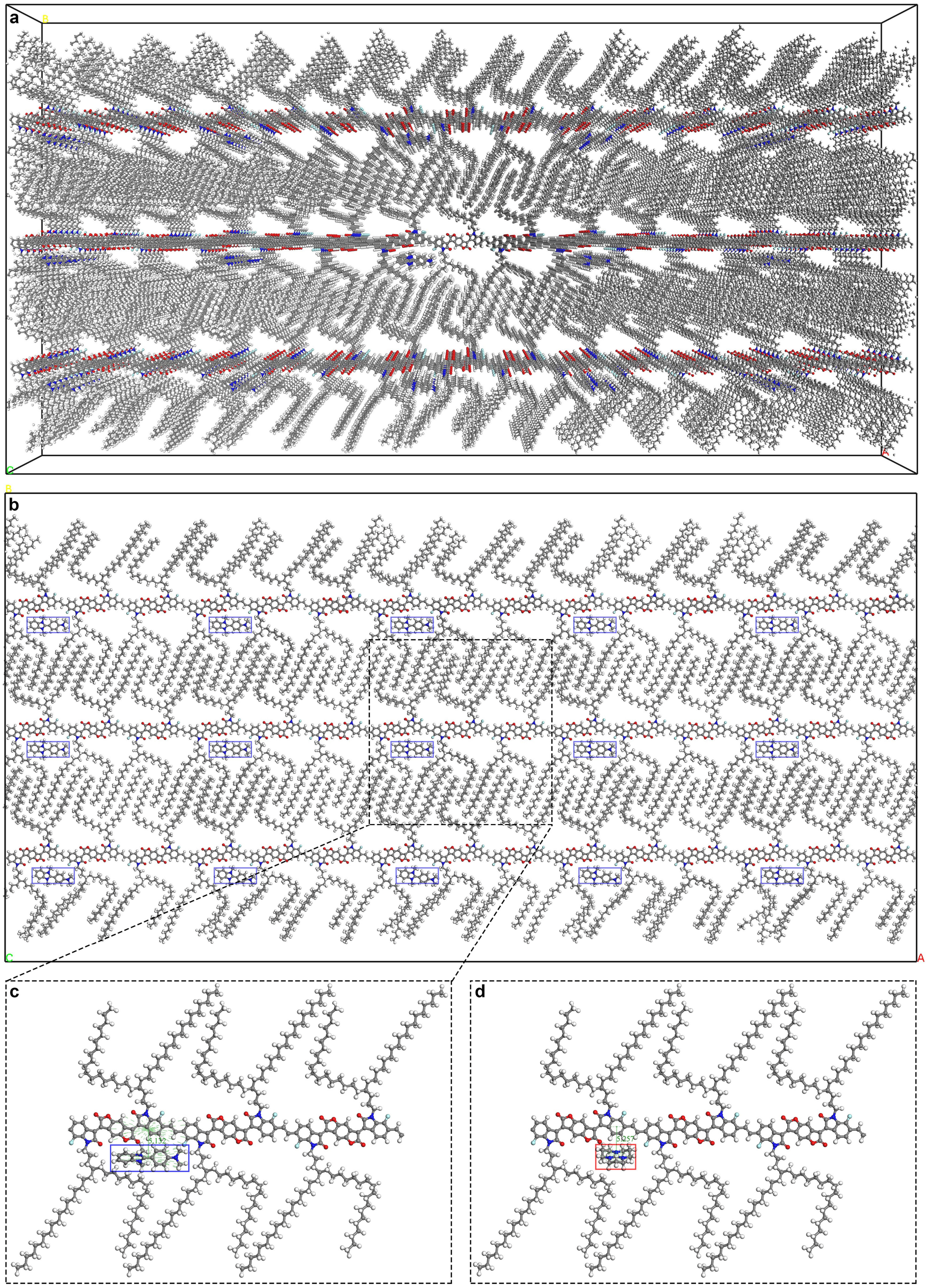

Supplementary Figure 6 | Initial configuration in molecular dynamics. a Perspective projection of the initial super cell. **b** Orthographic projection of the initial super cell. **c-d** Illustration of initial relative position of dopant cation and polymer anion in N-DMBI doped FBDPPV (**c**) or TAM doped FBDPPV (**d**). The unit in distance measurements is Å.

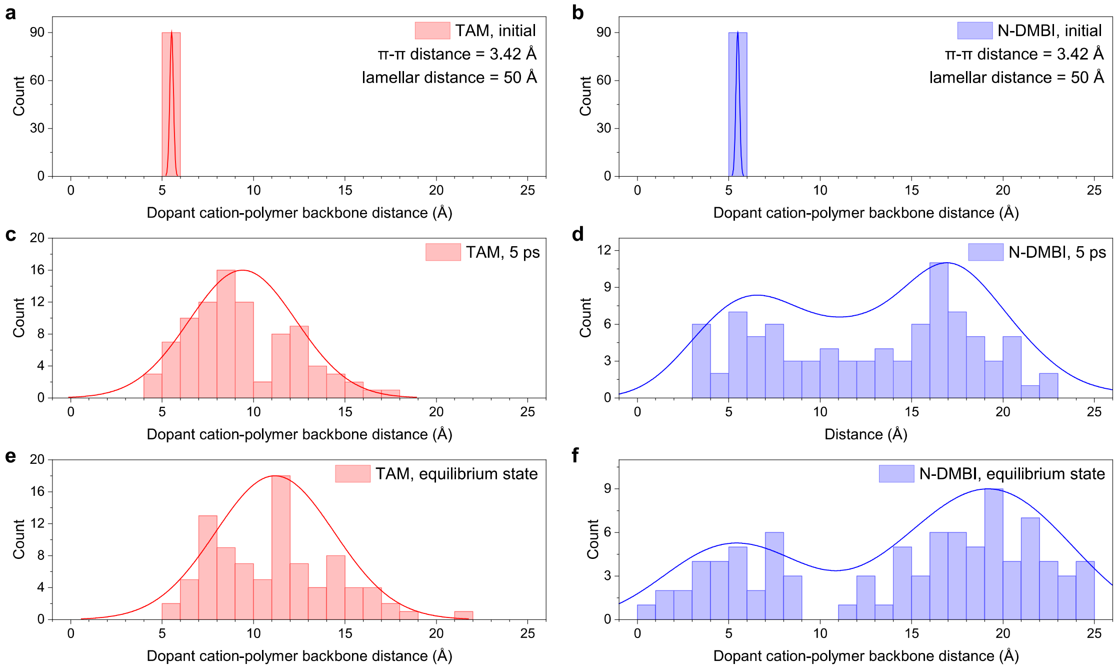

Supplementary Figure 7 | Cation-sidechain interaction prediction. a-f Histogram of distances between dopant cations and polymer conjugated backbones for TAM doped FBDPPV (**a**, **c**, **e**) and N-DMBI doped FBDPPV (**b**, **d**, **f**) with initial lamellar distance of 50 Å. Notice distance = 0 is polymer backbone and distance = 25 Å is alkyl chain tails.

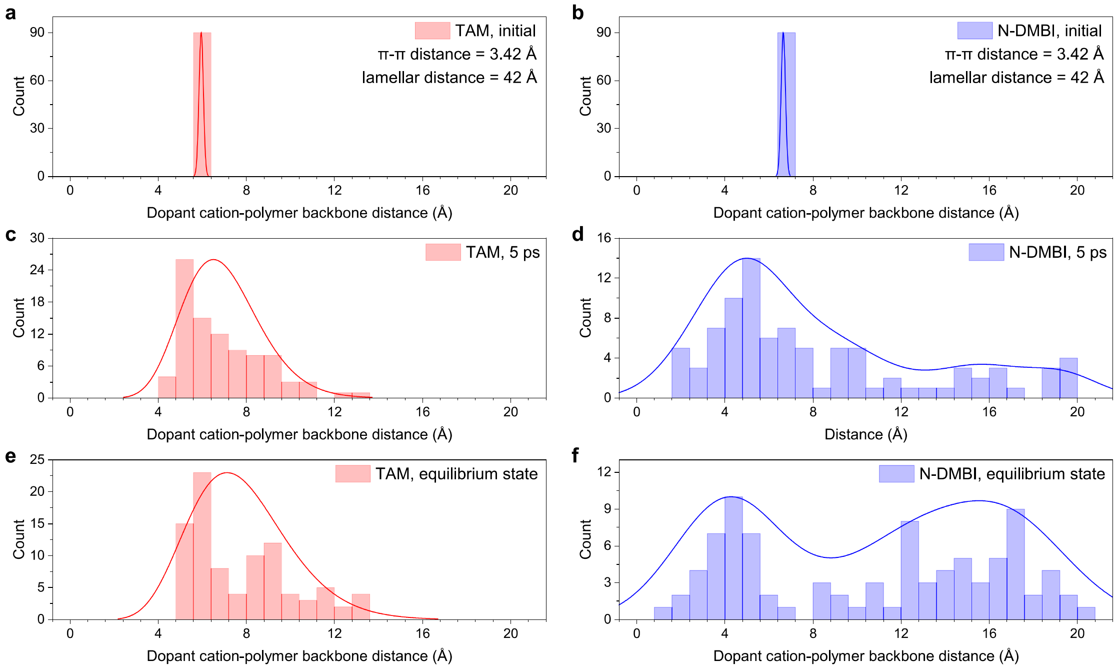

Supplementary Figure 8 | Cation-sidechain interaction prediction. a-f Histogram of distances between dopant cations and polymer conjugated backbones for TAM doped FBDPPV (**a**, **c**, **e**) and N-DMBI doped FBDPPV (**b**, **d**, **f**) with initial lamellar distance of 40 Å. Notice distance = 0 is polymer backbone and distance = 21 Å is alkyl chain tails. Statistical analysis shows that TAM^+ cations pervasively move away from the backbone toward middle of alkyl sidechains, while N-DMBI+ cations move toward the backbone or tails of alkyl sidechains, which is consistent with the results of 50 Å initial lamellar distance. This could imply that interactions between TAM^+/N-$DMBI^+$ cations and alkyl sidechains are inherent.

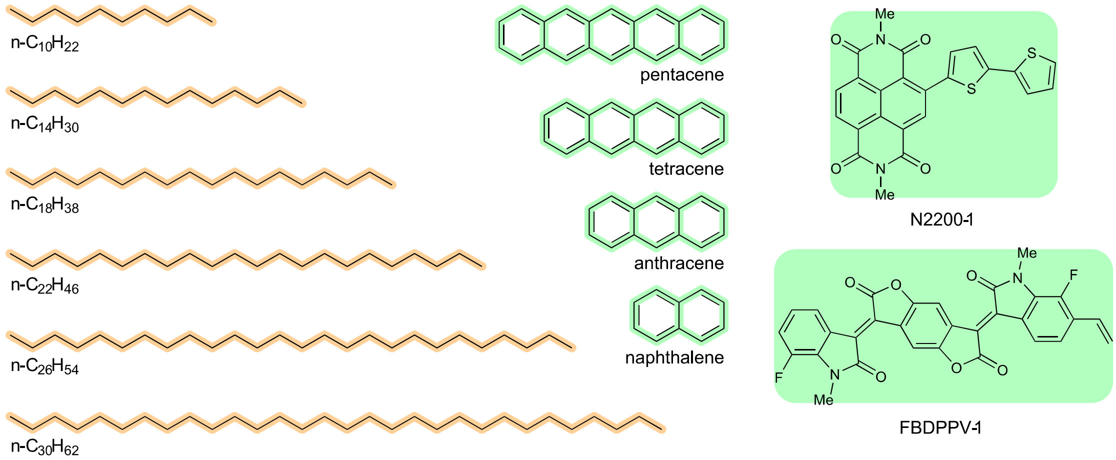

Supplementary Figure 9 | Molecular polarizability. Chemical structures of alkanes (n-$C_{10}H_{22}$, n-$C_{14}H_{30}$, n-$C_{18}H_{38}$, n-$C_{22}H_{46}$, n-$C_{26}H_{54}$, n-$C_{30}H_{62}$), arenes (naphthalene, anthracene, tetracene, pentacene), and monomer (N2200-1, PBDPPV-1) of N2200 and PBDPPV backbone.

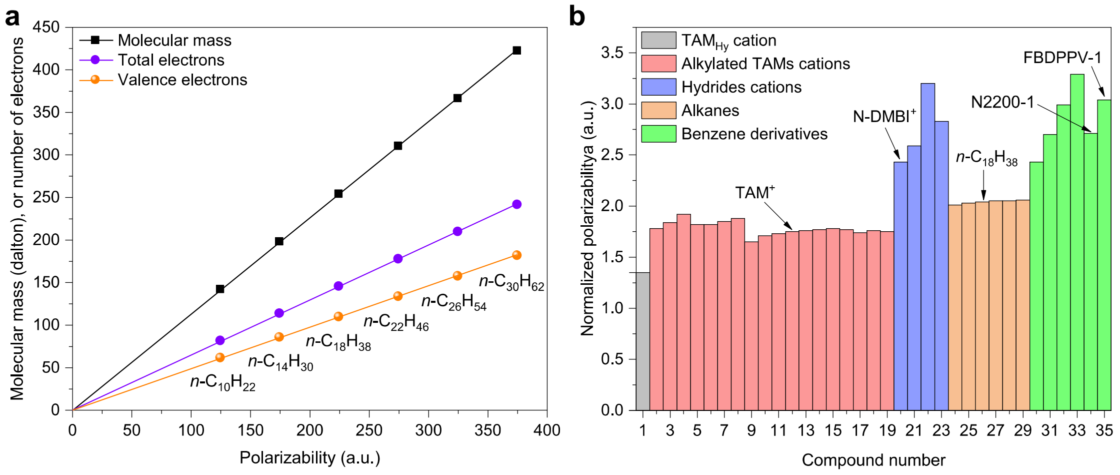

Supplementary Figure 10 | Molecular polarizability. a Relationship of molecular mass, number of total electrons, number of valance electrons, and polarizability in n-alkanes. **b** Normalized polarizability of $TAMs^+$ cations, cations of reported hydride dopants, n-alkanes, and benzene derivatives (including arenes and monomers). Compound numbers and molecular polarizability refer to Supplementary Table2. Polarizability are calculated under B3LYP/6-311+G(d,p) level.

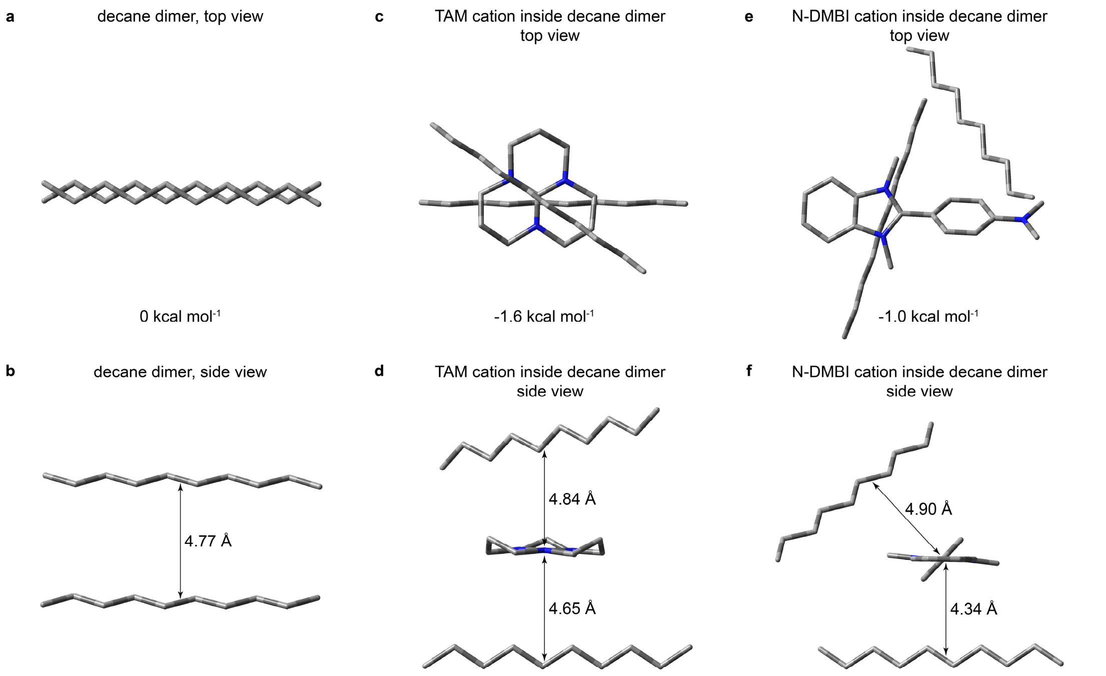

Supplementary Figure 11 | Cation-sidechain interactions. a-f Top view (**a**, **c**, **e**) and side view (**b**, **d**, **f**) of DFT optimized geometry structures of decane dimer (**a-b**), TAM cation inside decane dimer (**c-d**), and N-DMBI cation inside decane dimer (**e-f**), and their relative gibbs free energies (298K). DFT calculations performed under *ω*B97XD/6-311+G(d,p)//B3LYP/6-31G(d) level. TAM cation can form tight packing structures with alkyl chains and has stronger affinity with alkyl chains than N-DMBI cations.

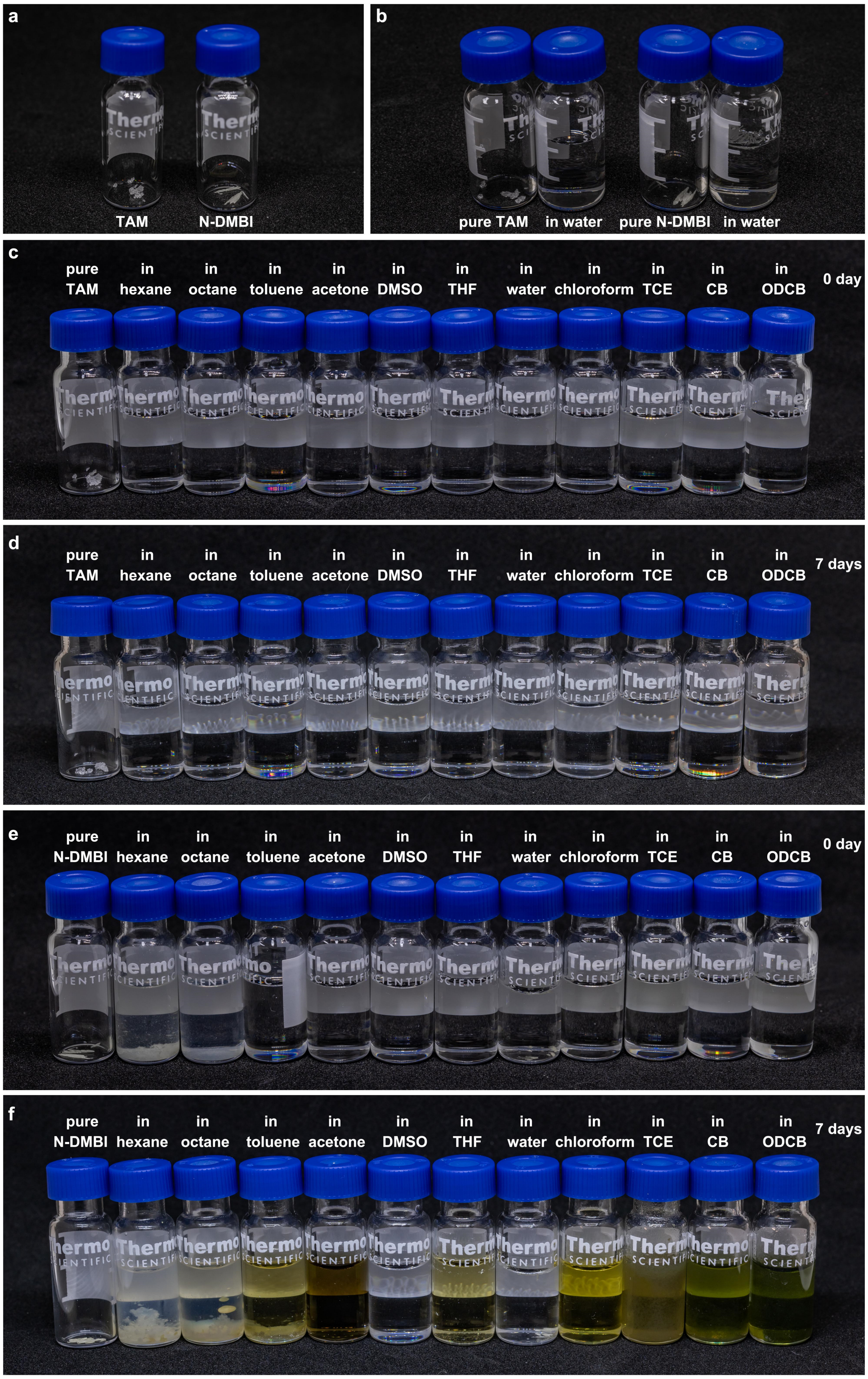

Supplementary Figure 12 | Solubility of TAM. a Photographs of TAM and N-DMBI. **b** Photographs of TAM, N-DMBI, and their solubleness in water. **c-f** solubleness and stability of TAM (**c** and **d**) and N-DMBI (**e** and **f**) in hexane, octane, toluene, acetone, DMSO, THF, water , chloroform, TCE, CB, ODCB (3 mg in 1 mL of solvent) in air for 0-7days. TAM shows good solubility and stability in all these solvents. N-DMBI shows poor solubility in alkanes and water, and is not stable in most solvents.

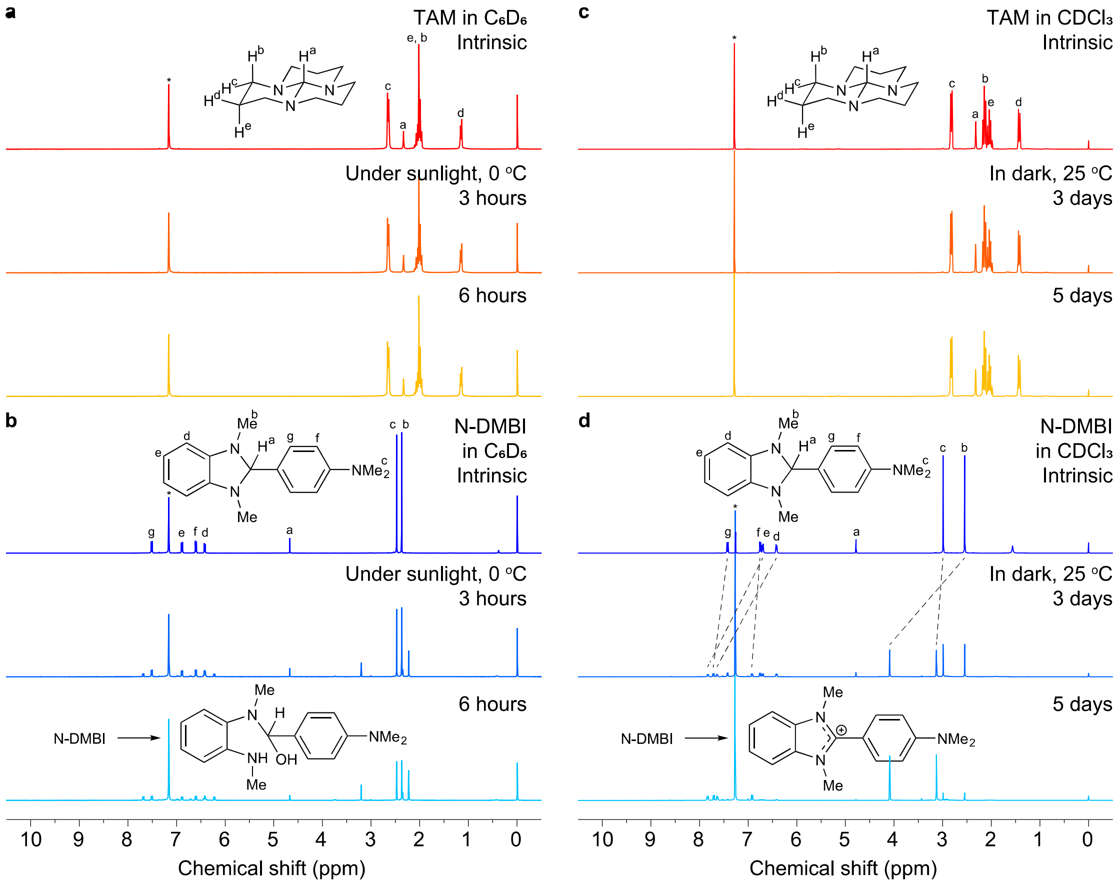

Supplementary Figure 13 | Chemical stability. a-b Time-dependent ^{1}H NMR spectra of TAM (**a**) and N-DMBI (**b**) in benzene-d6 under sunlight. **c-d** Time-dependent ^{1}H NMR of TAM (**c**) and N-DMBI (**d**) in chloroform-d in dark. TAM is stable in ether chlorinated solvent and under light, while N-DMBI is not stable in these conditions.

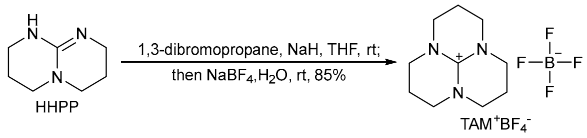

Supplementary Figure 14 | Synthesis of TAM$^+$ cation. Synthesis of TAM cantion is similar to synthesis of TAM except the reduction.

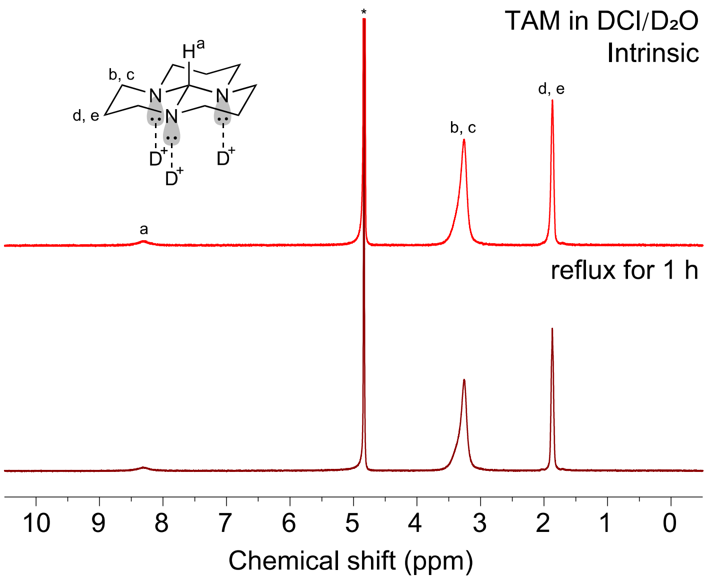

Supplementary Figure 15 | Chemical stability. Time-dependent ^{1}H NMR spectra of TAM in saturated DCl-*d* (D_2O-d_2 solution). Notice that ^{1}H NMR spectra of TAM are totally different from that of TAM^+BF^{4-} in the same conditions (See Part 9. ^{1}H and ^{13}C NMR spectra), indicating that TAM cannot transfer hydride to proton to generate hydrogen in boiling hydrochloric acid. Therefore, TAM is stable in boiling hydrochloric acid.

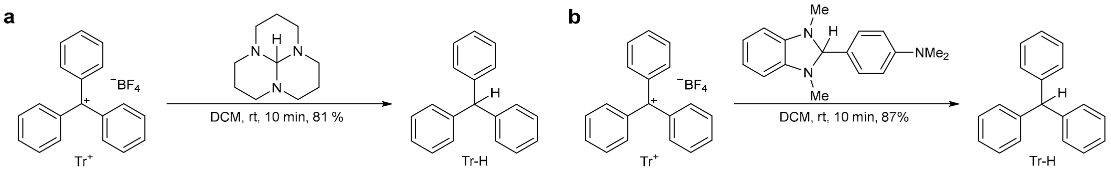

Supplementary Figure 16 | Hydride-transfer reactions. Both TAM and N-DMBI can transfer hydride to strong electrophile in high yield.

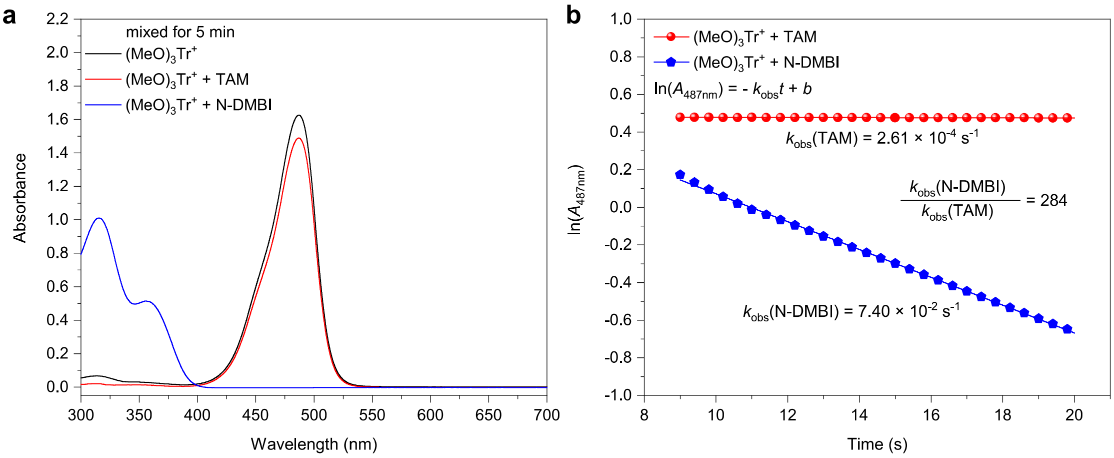

Supplementary Figure 17 | Hydride-transfer reaction rates. a Absorption spectra of intrinsic and TAM (or N-DMBI) mixed $(MeO)_3Tr^+$ after mixing for 5 min. **b** Time-dependent absorption of hydride-transfer reaction between TAMor N-DMBI and tris(4-methoxyphenyl)methylium tetrafluorobate ($(MeO)_3Tr^+$). The initial concentrations are 2.38×10^{-5} M for $(MeO)_3Tr^+$, 1.42×10^{-4} M for both TAM and N-DMBI in anhydrous dichloromethane.

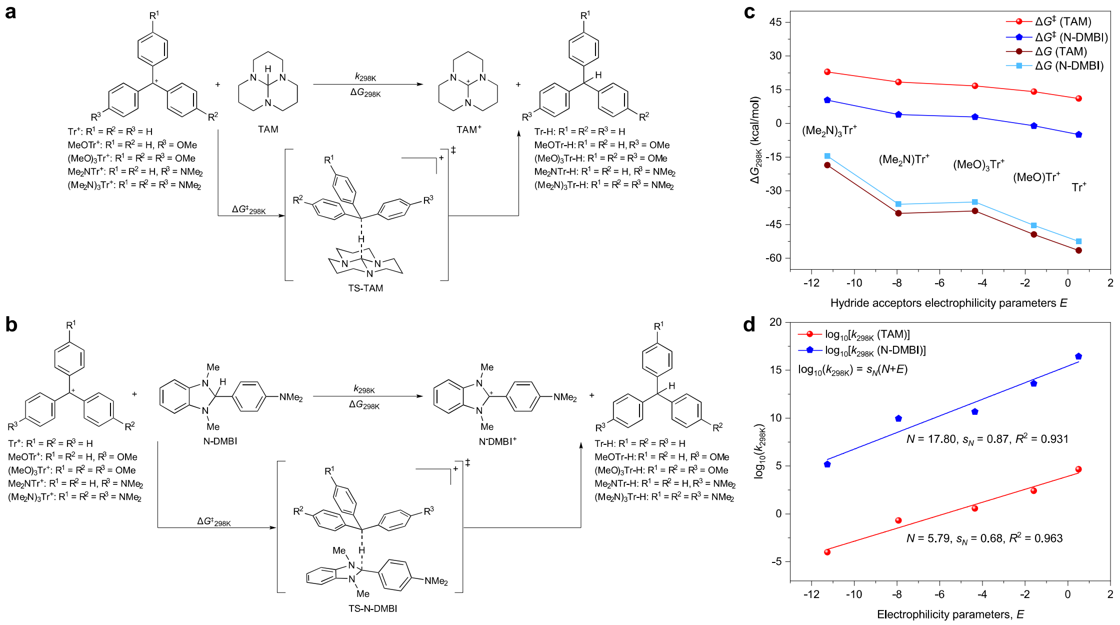

Supplementary Figure 18 | Kinetic nucleophilicity. a-b Hydride-transfer reactions between TAM (**a**) or N-DMBI (**b**) and substituted tritylium ions with various electrophilicities. **c** DFT calculated activation Gibbs free energy ($\Delta G^{\ddagger}$) and reaction Gibbs free energy (ΔG) of the hydride-transfer reactions. **d** DFT calculated rate constant of the hydride-transfer reactions, kinetic hydride nucleophilicity (N) and sensitivity parameter (s_N) of TAM and N-DMBI. DFT calculations performed under ωB97XD/6-311+G(d,p)//B3LYP/6-31G(d) level.

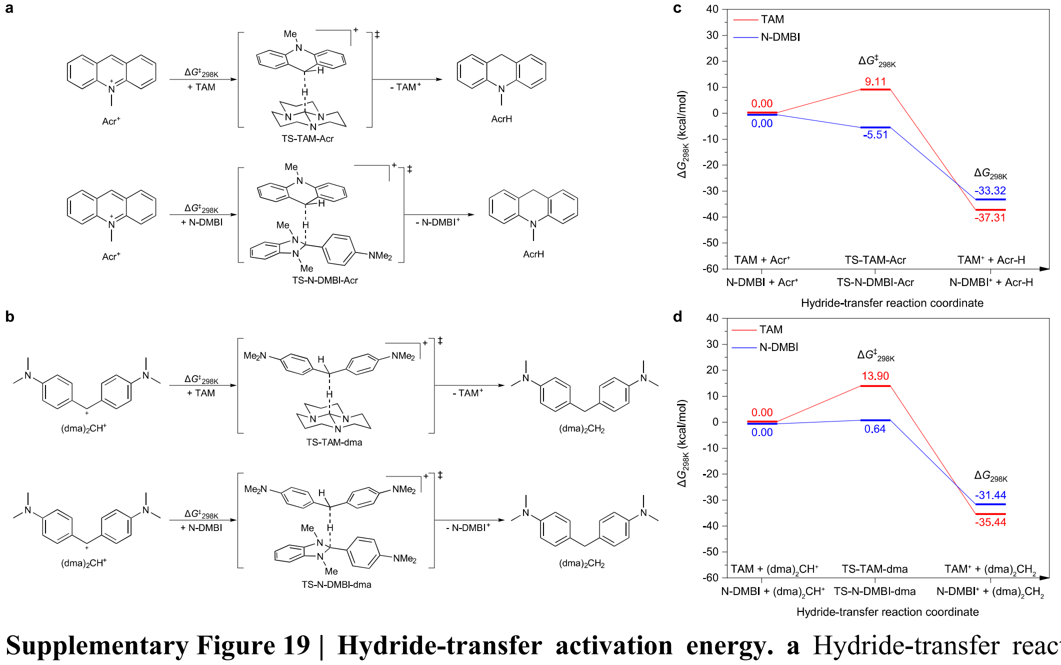

Supplementary Figure 19 | Hydride-transfer activation energy. a Hydride-transfer reactions between TAM or N-DMBI and bis(4-(dimethylamino)phenyl)methylium ($(dma)_2CH^+$). **b** Hydride-transfer reactions between TAM or N-DMBI and 10-methylacridinium (Acr^+). **c** DFT calculated energy surface for hydride-transfer reactions with $(dma)_2CH^+$. **d** DFT calculated energy surface for hydride-transfer reactions with Acr^+. DFT calculations performed under ωB97XD/6-311+G(d,p)//B3LYP/6-31G(d) level. TAM shows higher activation Gibbs free energy (also 12~16 kcal/mol higher) in hydride-transfer reactions with other typical electrophiles.

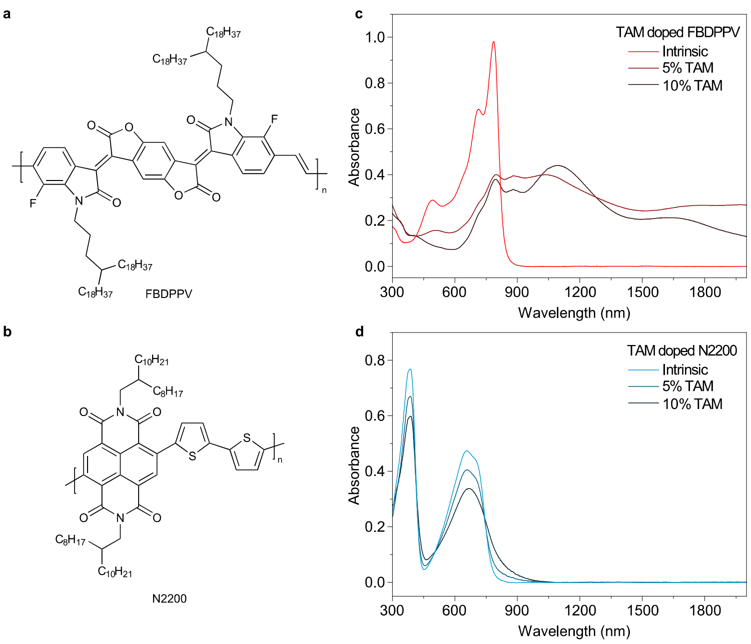

Supplementary Figure 20 | Absorption spectra of doped semiconductors. a-b Chemical structure of FBDPPV and N2200. **c-d** UV-vis-NIR absorption spectra of intrinsic and TAM doped FBDPPV (**c**) and N2200 (**d**) in dilute ODCB solution after heating in 140 °C for 5 min. The polymer concentration keeps 20 mg/L (in ODCB) for each case, with different mass fraction of TAM. TAM can n-dope FBDPPV and N2200 in high temperature.

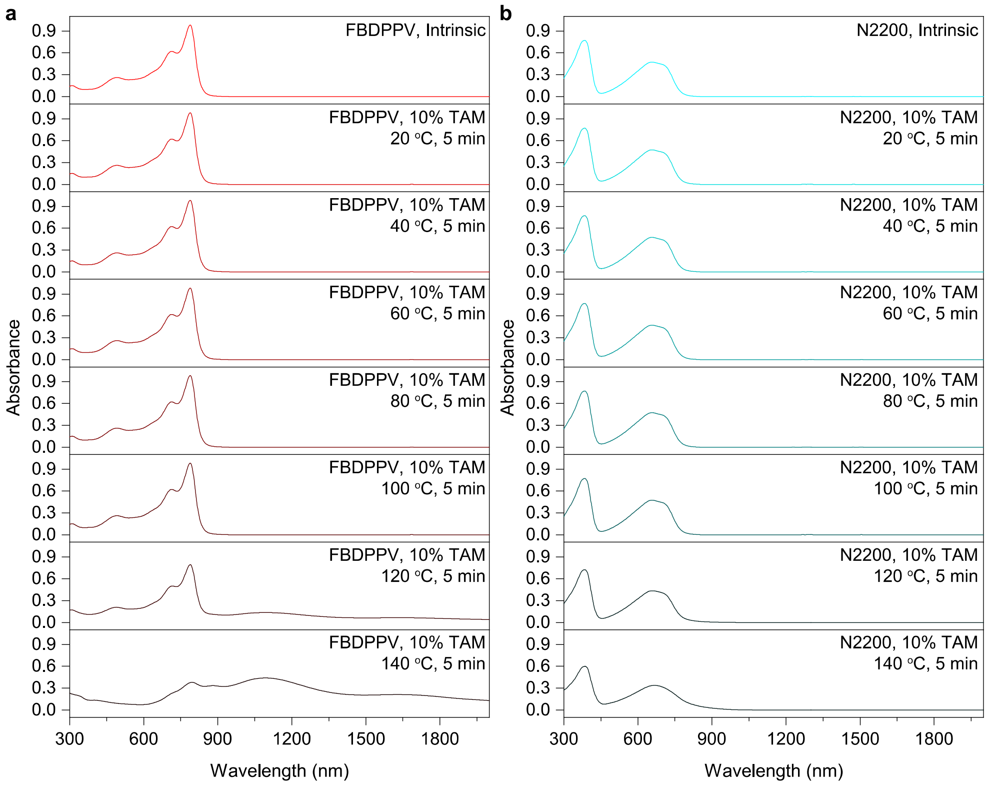

Supplementary Figure 21 | Absorption spectra of doped semiconductors. a-b Temperature-dependent UV-vis-NIR absorption spectra of TAM doped FBDPPV (**a**) and N2200 (**b**) in dilute ODCB solution. The polymer concentration keeps 20 mg/L for each case. TAM can n-dope FBDPPV and N2200 in only high temperature.

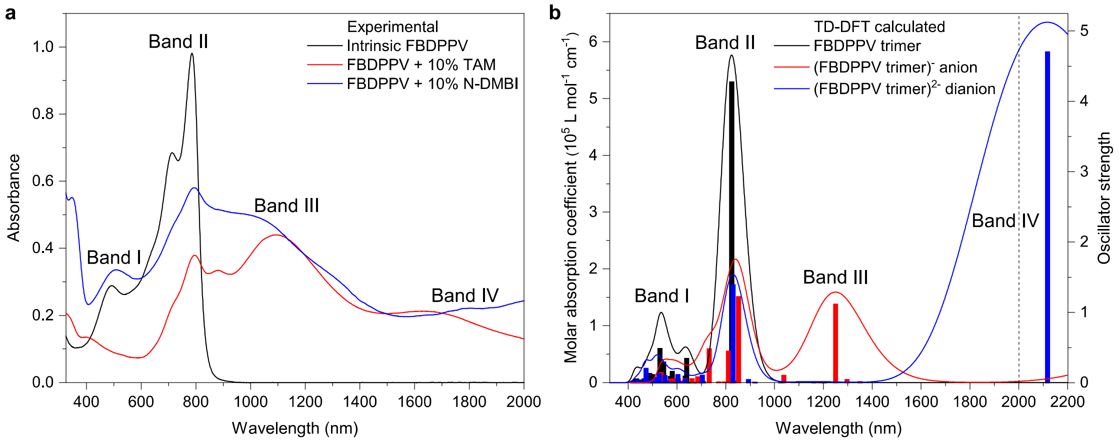

Supplementary Figure 22 | Experimental and calculated absorption spectra. a Experimental absorption spectra of intrinsic and doped FBDPPV in dilute ODCB solution at room temperature. The FBDPPV concentration keeps 20 mg/L for each case, after mixing with TAM (at 140 ºC for 5 min) or N-DMBI (at room temperature for 5 min). **b** Time-dependent density functional theory (TD-DFT) calculated absorption spectra and oscillator strength of FBDPPV trimer, (FBDPPV trimer)$^{-}$ anion, and (FBDPPV trimer)$^{2-}$ dianion.

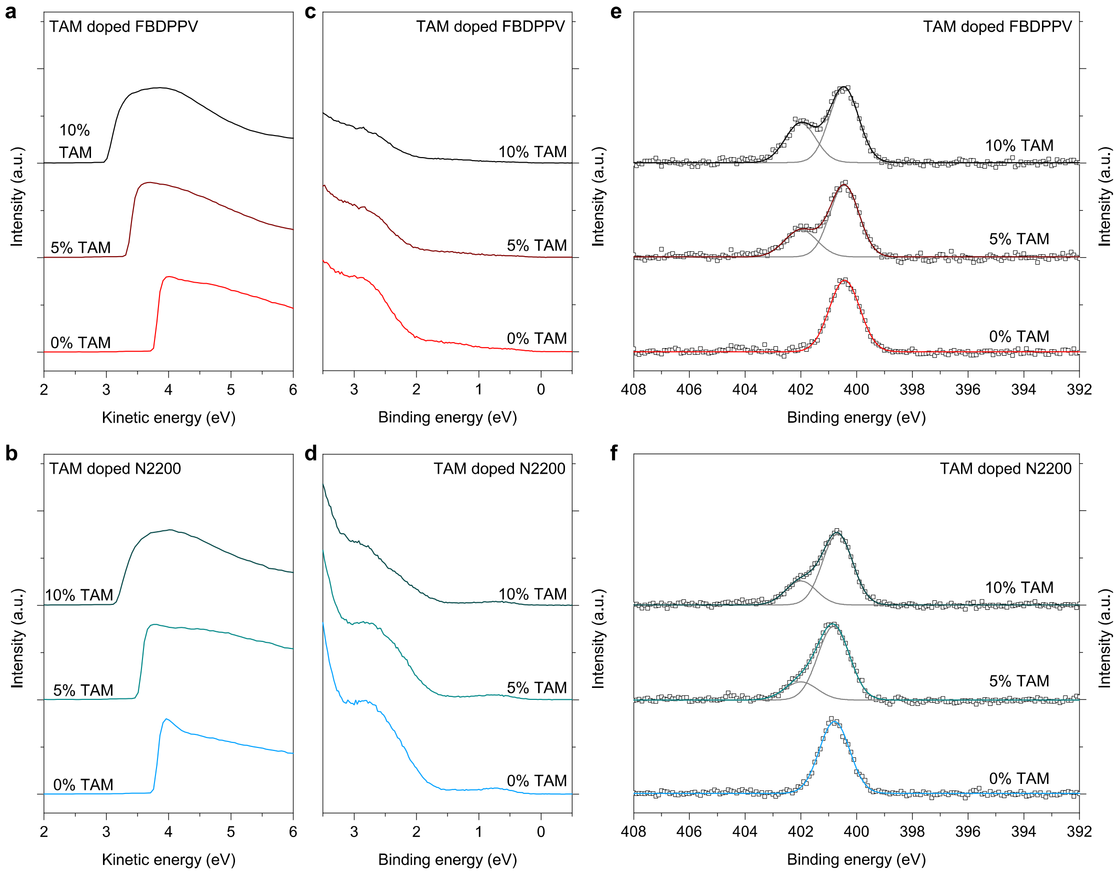

Supplementary Figure 23 | Doping ability. a-d Ultraviolet photoelectron spectra of intrinsic and TAM doped FBDPPV (**a**, **c**) and N2200 (**b**, **d**) thin films at low kinetic energy region (**a-b**) and low binding energy region (**c-d**). **e-f** N (1s) X-ray photoelectron spectra of intrinsic and TAM doped FBDPPV (**c**) and N2200 (**d**) in thin films. TAM can effectively n-dope polymers.

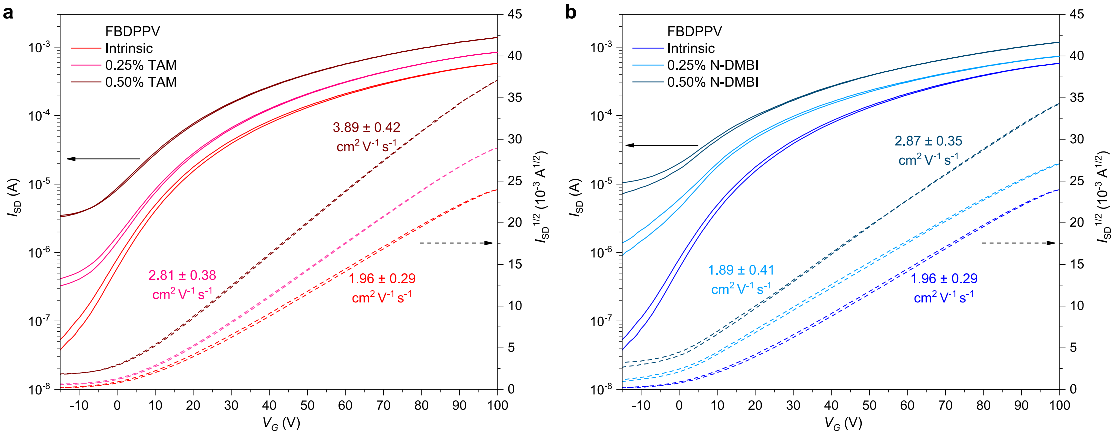

Supplementary Figure 24 | Doping ability. a-b Transfer characteristics of intrinsic and TAM doped FBDPPV (**a**) or N-DMBI doped FBDPPV (**b**) based field-effect transistors (FETs). FET measurements suggest that both TAM and N-DMBI doping can enhance electron mobility of FBDPPV, and this effect is more pronounced in TAM than N-DMBI.

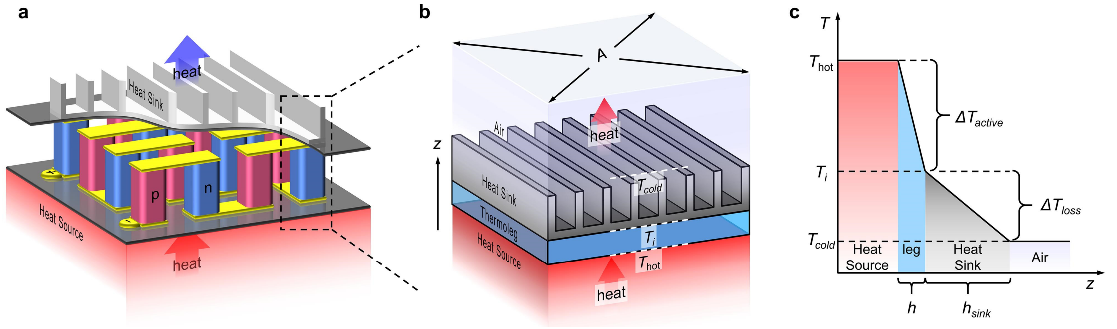

Supplementary Figure 25 | Influence of thermoleg thickness on vertical device performance. a Schematic diagram of vertical thermoelectric generator. **b** Heat transport in vertical thermoelectric generator with single thermoleg. **c** Temperature distribution in vertical thermoelectric generator with single thermoleg. The thermoleg thickness affects temperature distribution and effective temperature difference.

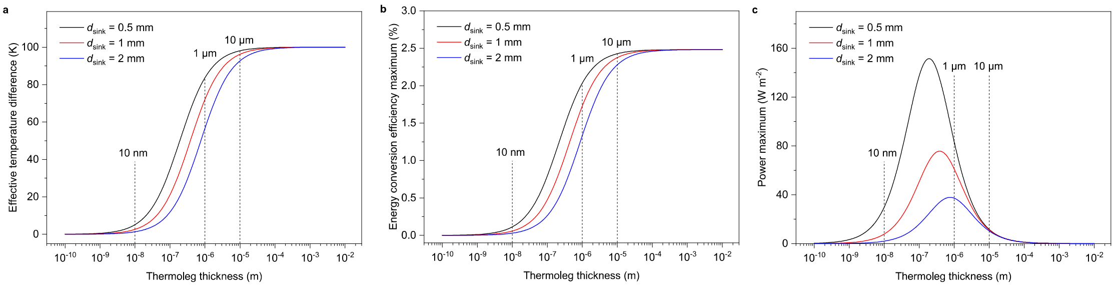

Supplementary Figure 26 | Influence of thermoleg thickness on vertical device performance. a Thickness-dependent effective temperature difference. **b** Thickness-dependent maximum PCE. **c** Thickness-dependent maximum output power. The vertical thermoelectric generator with thick thermolegs present much enhanced maximum PCE and output power than thin thermolegs.

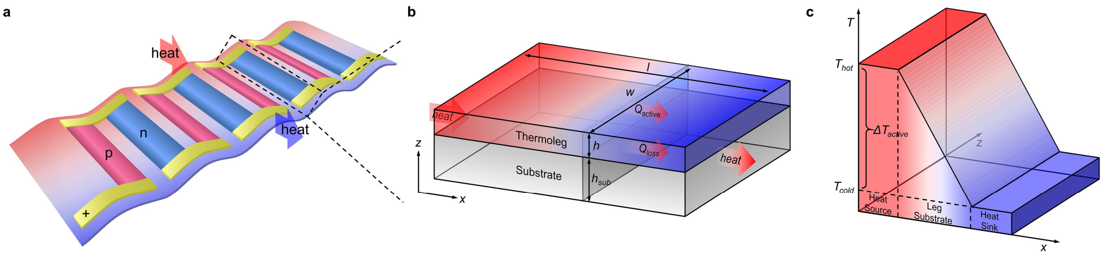

Supplementary Figure 27 | Influence of thermoleg thickness on lateral device performance. a Schematic diagram of lateral thermoelectric generator. **b** Heat transport in lateral thermoelectric generator with single thermoleg. **c** Temperature distribution in lateral thermoelectric generator with single thermoleg. The thermoleg thickness affects heat transfer efficiency.

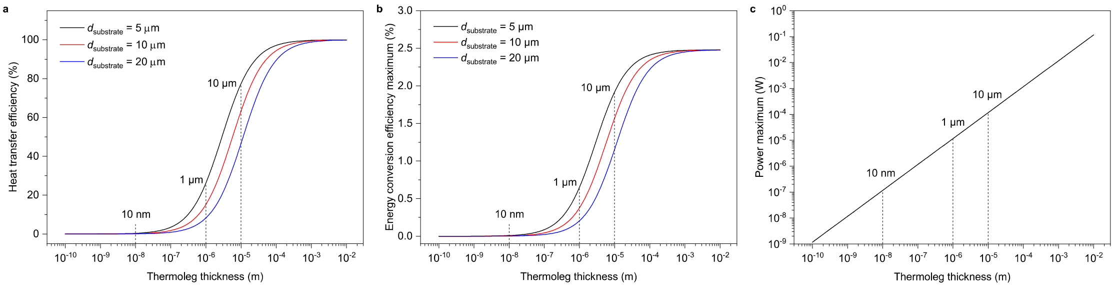

Supplementary Figure 28 | Influence of thermoleg thickness on lateral device performance. a Thickness-dependent heat transfer efficiency. **b** Thickness-dependent maximum PCE. **c** Thickness-dependent maximum output power. The lateral thermoelectric generator with thick thermolegs present greatly enhanced maximum PCE and output power than thin thermolegs.

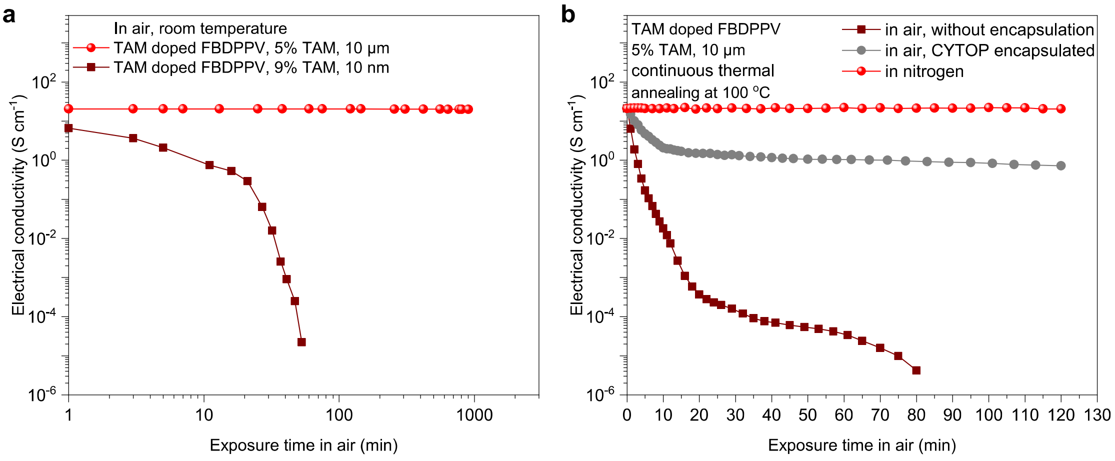

Supplementary Figure 29 | Stability of electrical conductivity. a Comparison of electrical conductivity stability between TAM-doped FBDPPV thin film (10 nm) and thick film (10 μm) without encapsulation (at their electrical conductivity maxima, under ambient conditions: 25 °C, R_H = 50~60%). **b** Time-dependent electrical conductivity of TAM-doped FBDPPV thick film under long-term continuous annealing.

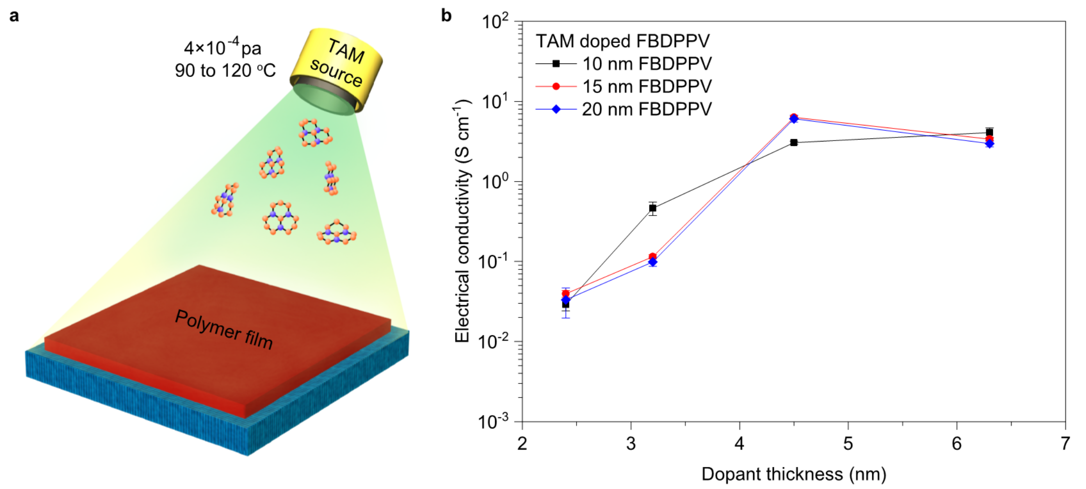

Supplementary Figure 30 | Vapor doping. **a** Scheme of TAM vapor doping method. **b** Electrical conductivity of TAM vapor doped FBDPPV in different polymer and dopant thickness. Error bars indicate the s.d. of ten experimental replicates. TAM vapor doped FBDPPV thin film could obtain electrical conductivity over 6 S cm^{-1}.

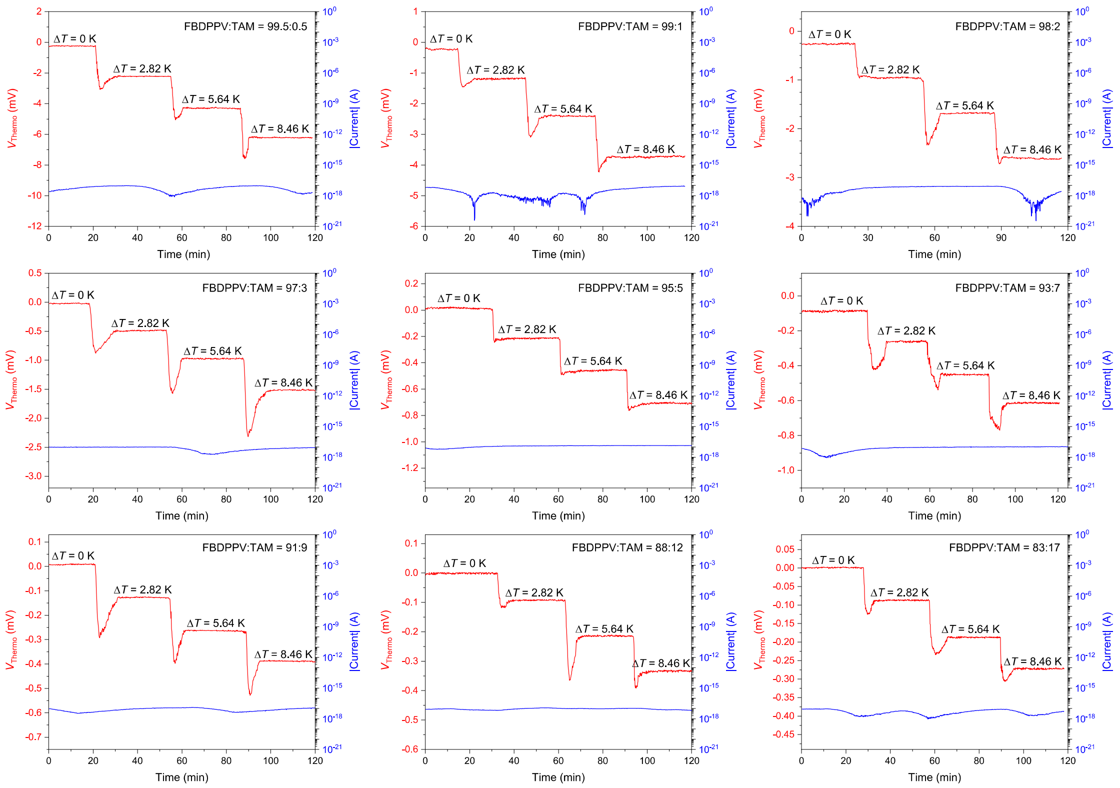

Supplementary Figure 31 | Seebeck coefficient measurements. Temperature difference dependent thermal voltage and current in thermal voltage measurement for TAM doped FBDPPV in thickness of 10 μm.

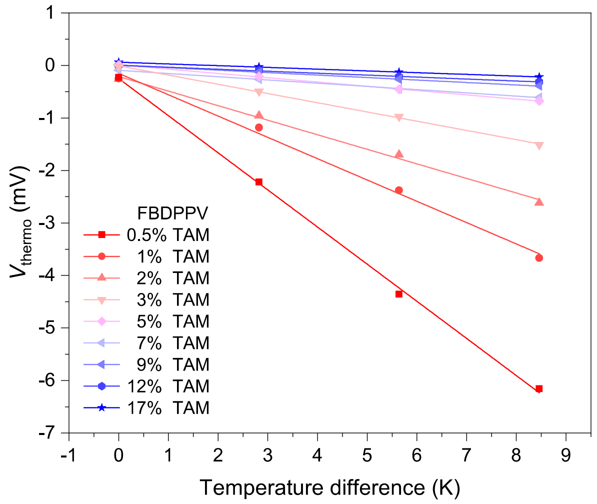

Supplementary Figure 32 | Seebeck coefficient measurements. Temperature difference dependent thermal voltage of TAM doped FBDPPV in thickness of 10 μm.

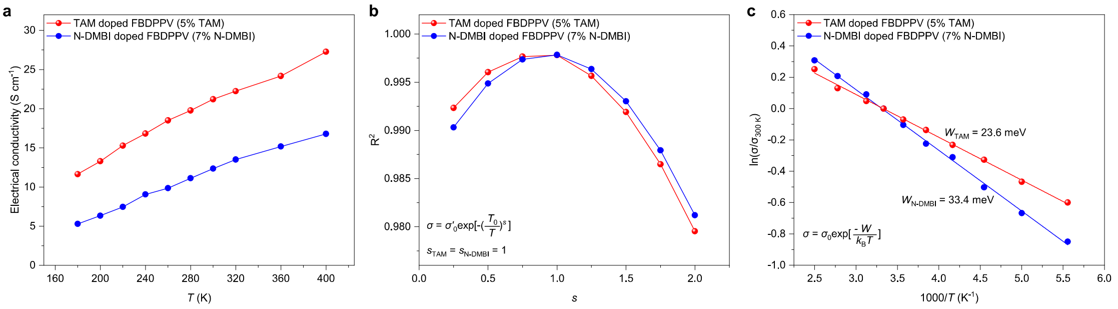

Supplementary Figure 33 | Temperature dependent electrical conductivity. a Temperature dependent electrical conductivity of TAM/N-DMBI doped FBDPPV (at their electrical conductivity maxima). **b** Evaluation of the nearest-neighbor hopping (NNH) conduction exponent. **c**, Activation energy (W) of TAM/N-DMBI doped FBDPPV in NNH conduction.

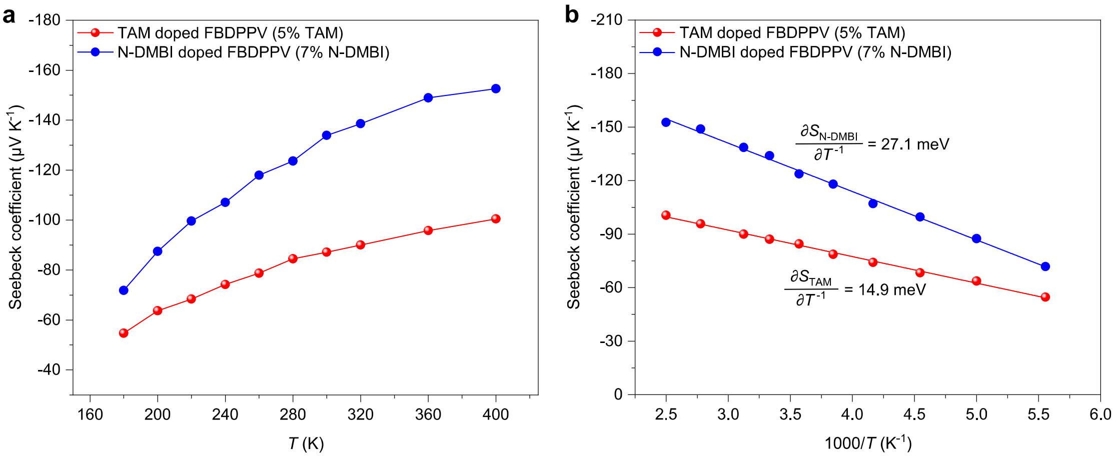

Supplementary Figure 34 | Temperature dependent Seebeck coefficient. a-b Temperature dependent Seebeck coefficient of TAM/N-DMBI doped FBDPPV (at their electrical conductivity maxima).

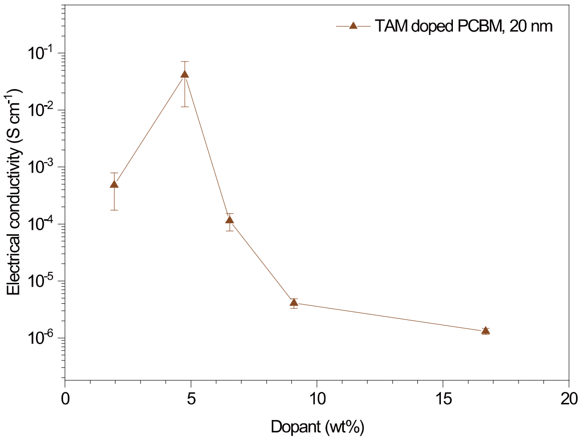

Supplementary Figure 35 | TAM doped small molecule semiconductor. Electrical conductivity of TAM doped $PC_{61}BM$. Error bars indicate the s.d. of ten experimental replicates. TAM doped $PC_{61}BM$ films have electrical conductivity up to 0.04 S cm^{-1}.

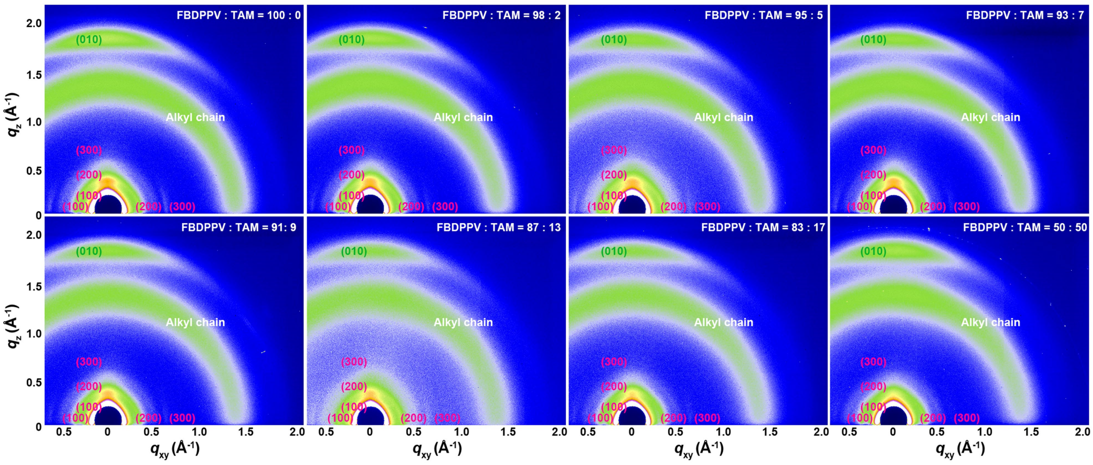

Supplementary Figure 36 | GIWAXS analysis. 2D GIWAXS patterns of intrinsic and TAM doped FBDPPV.

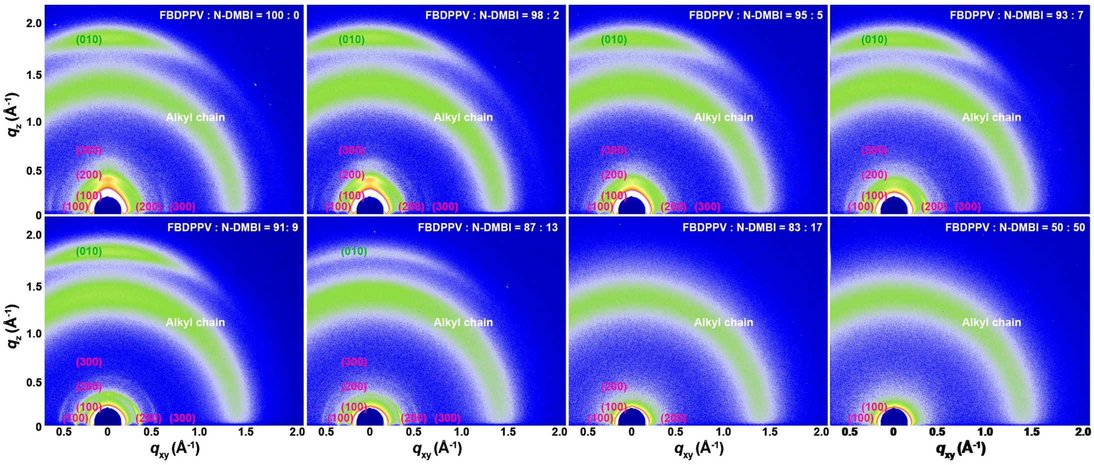

Supplementary Figure 37 | GIWAXS analysis. 2D GIWAXS patterns of intrinsic and N-DMBI doped FBDPPV.

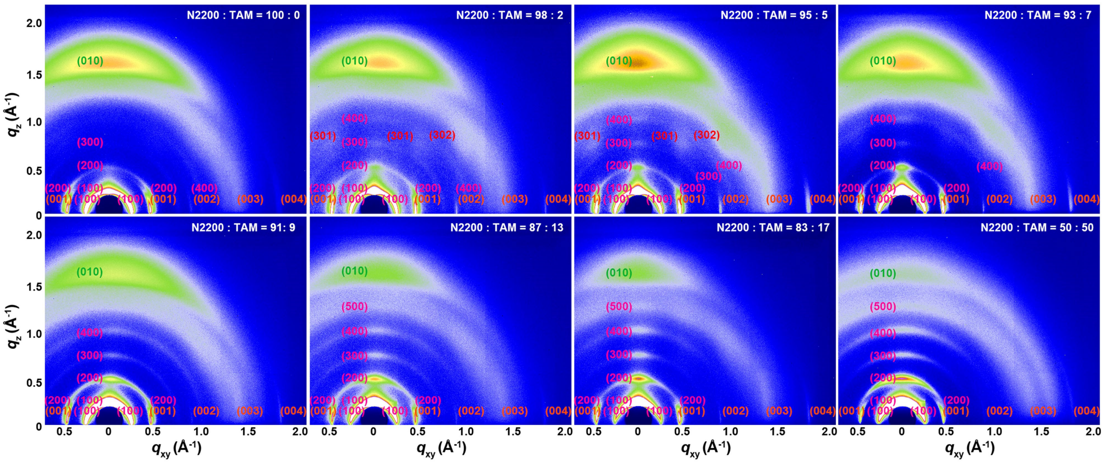

Supplementary Figure 38 | GIWAXS analysis. 2D GIWAXS patterns of intrinsic and TAM doped N2200.

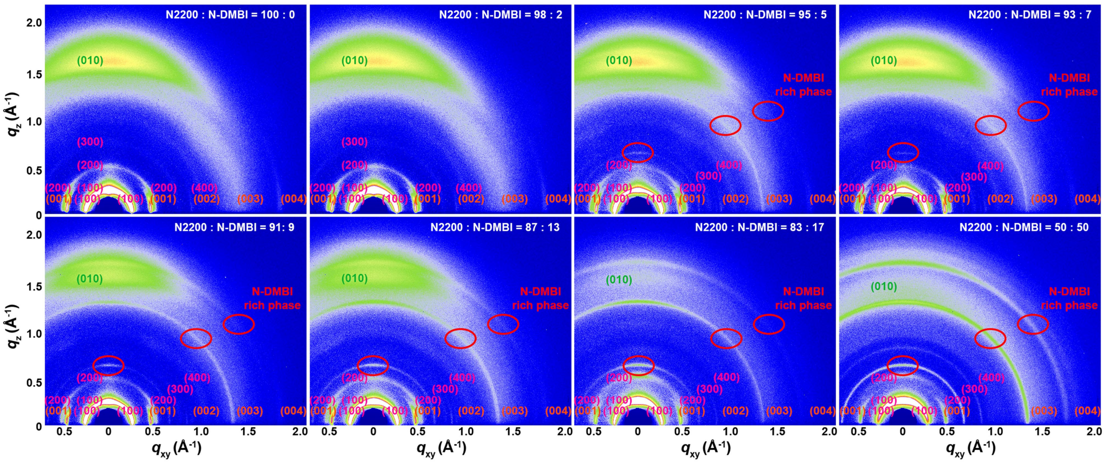

Supplementary Figure 39 | GIWAXS analysis. 2D GIWAXS patterns of intrinsic and N-DMBI doped N2200.

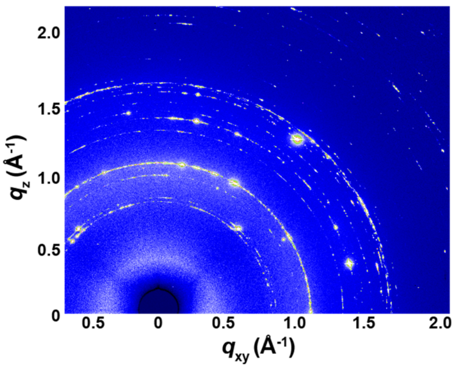

Supplementary Figure 40 | GIWAXS analysis. 2D GIWAXS patterns of TAM thin films (drop-casted from 3 mg/mL *o*-DCB solution)

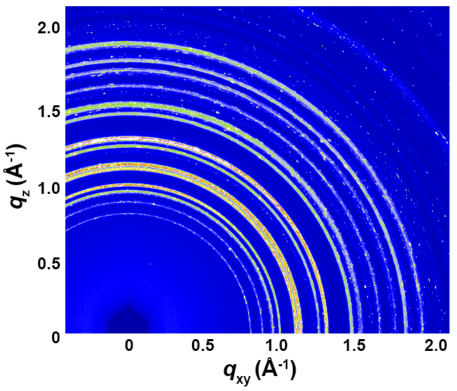

Supplementary Figure 41 | GIWAXS analysis. 2D GIWAXS patterns of N-DMBI thin films (drop-casted from 3 mg/mL *o*-DCB solution)

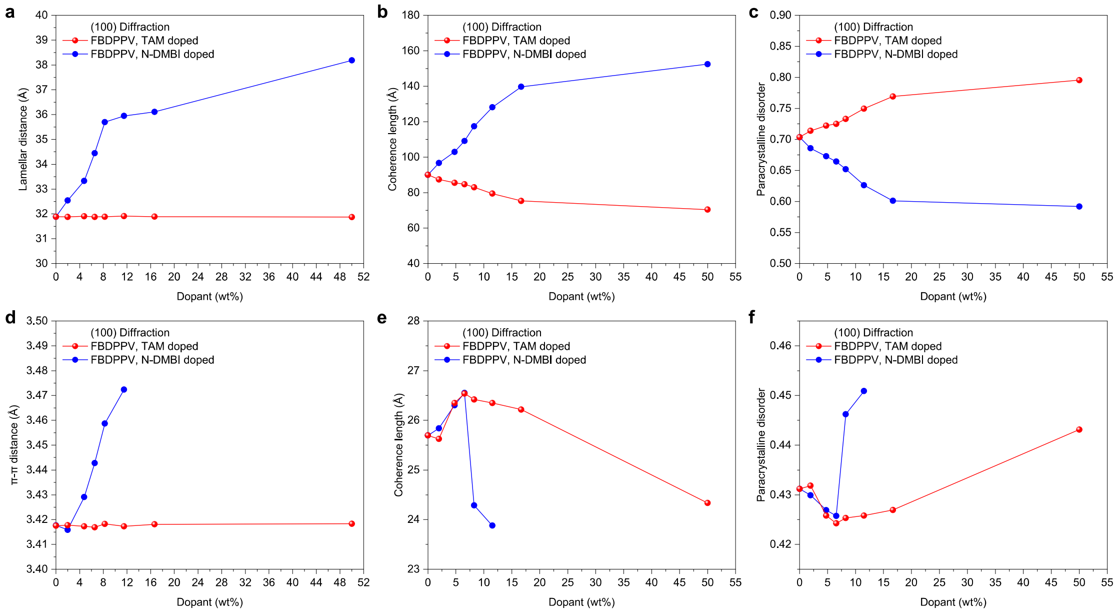

Supplementary Figure 42 | GIWAXS analysis of intrinsic and TAM/N-DMBI doped FBDPPV. a-c Lamellar distances (**a**), coherence length (**b**), and paracrystalline disorder analysis. (**c**). **d-f** π-π distance (**d**), coherence length (**b**), and paracrystalline disorder analysis.

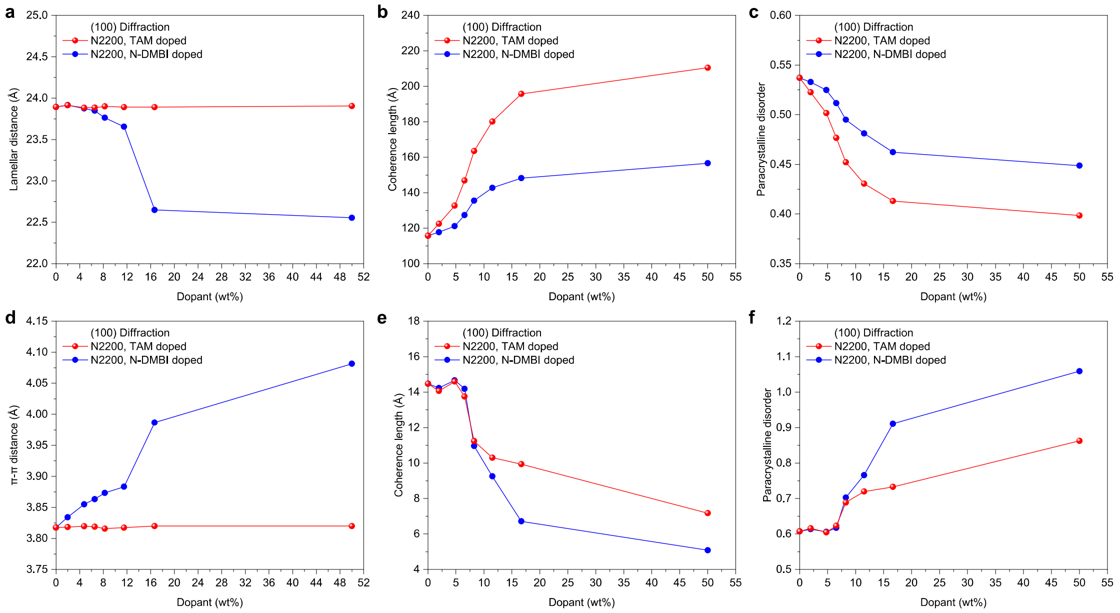

Supplementary Figure 43 | GIWAXS analysis of intrinsic and TAM/N-DMBI doped N2200. a-c Lamellar distances (**a**), coherence length (**b**), and paracrystalline disorder analysis. (**c**). **d-f** π-π distance (**d**), coherence length (**b**), and paracrystalline disorder analysis.

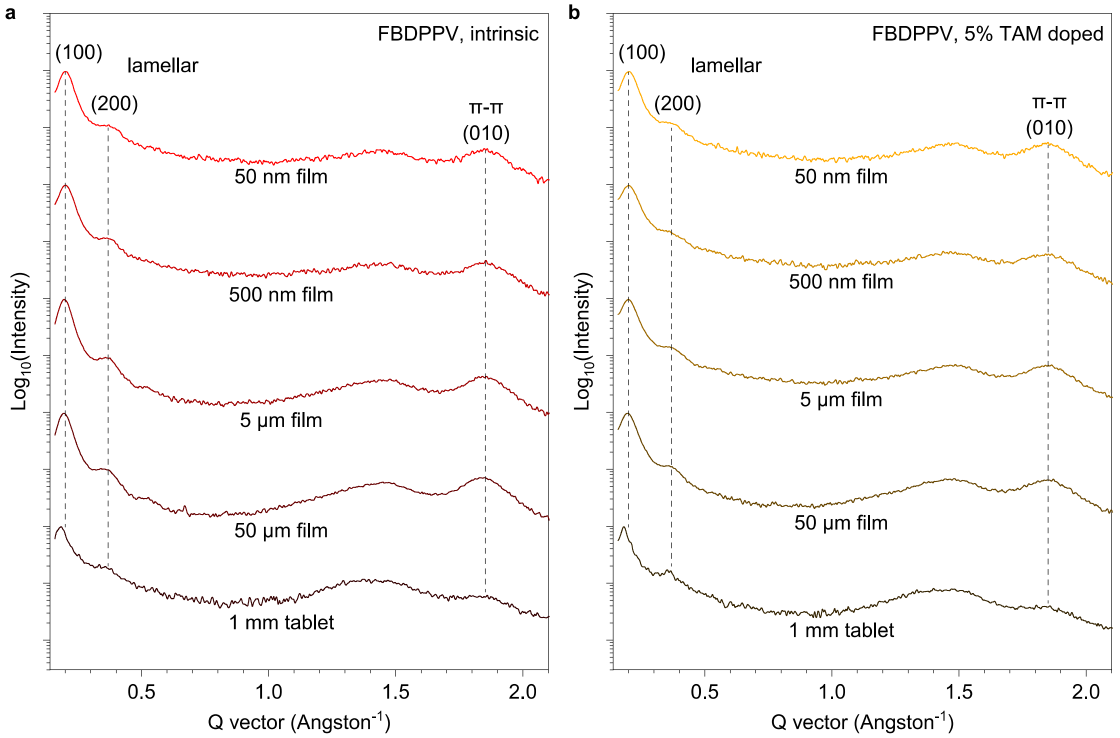

Supplementary Figure 44 | GIWAXS analysis. a-b Out-of-plane GIWAXS analysis of intrinsic (**a**) and TAM doped (**b**) FBDPPV in thin films, thick films, and tablet states. FBDPPV and TAM doped FBDPPV present nearly thickness-independent molecular packing.

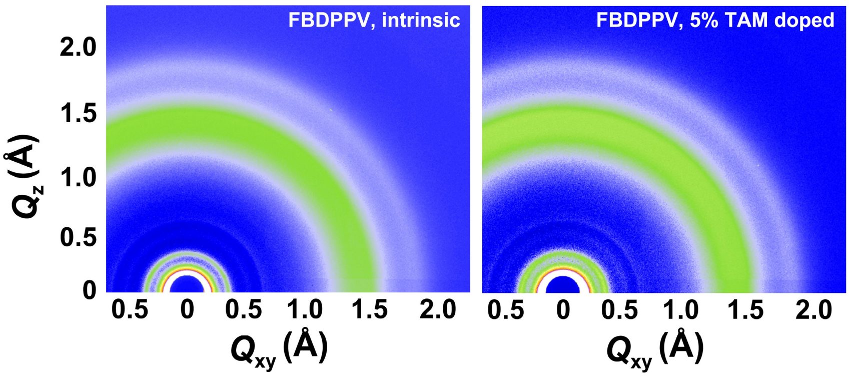

Supplementary Figure 45 | GIWAXS analysis. 2D GIWAXS patterns of intrinsic and TAM doped FBDPPV in 1 mm tablet states.

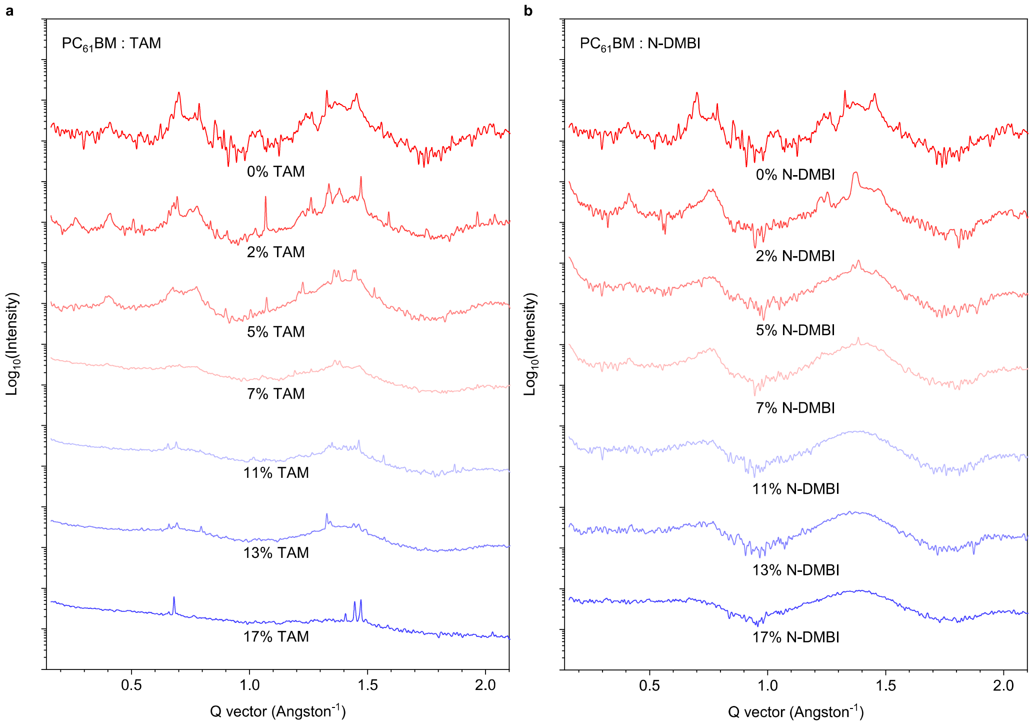

Supplementary Figure 46 | GIWAXS analysis. a-b Out-of-plane GIWAXS analysis of intrinsic and doped $PC_{61}BM$: TAM doped $PC_{61}BM$ (**a**), N-DMBI doped $PC_{61}BM$ (**b**).

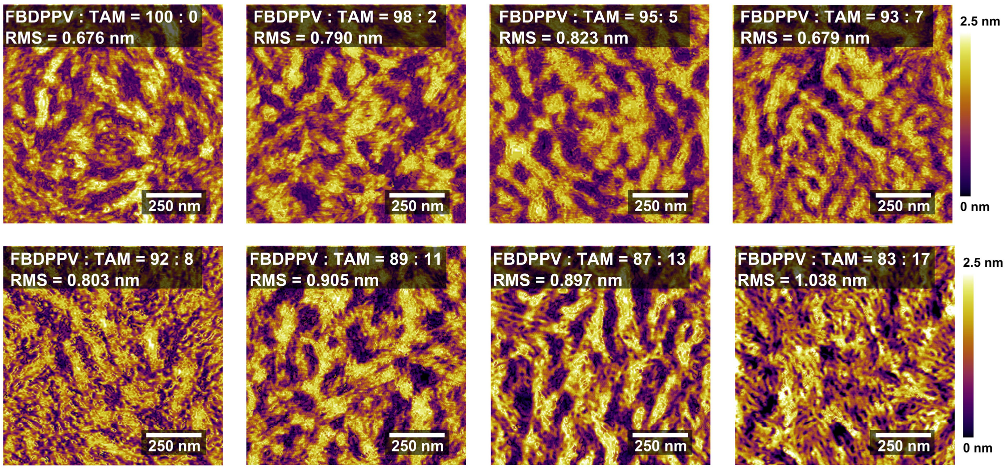

Supplementary Figure 47 | AFM analysis. AFM height images of intrinsic and TAM doped FBDPPV.

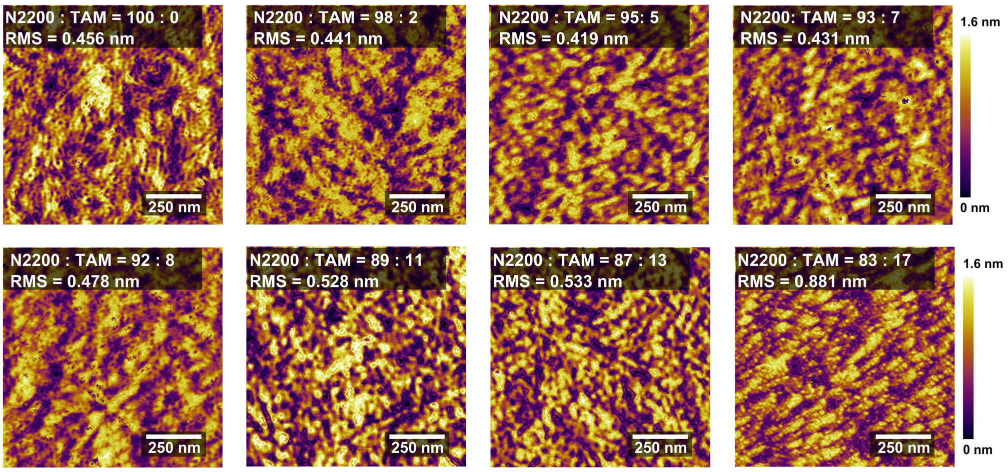

Supplementary Figure 48 | AFM analysis. AFM height images of intrinsic and TAM doped N2200.

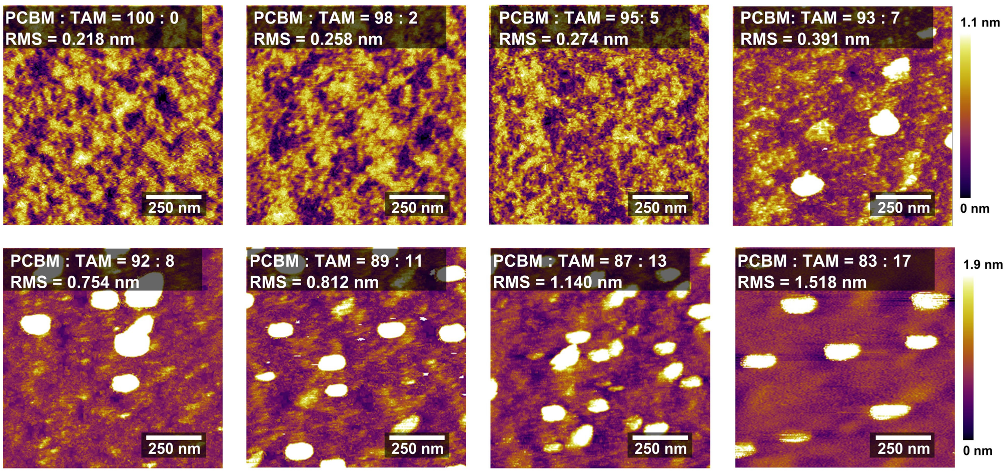

Supplementary Figure 49 | AFM analysis. AFM height images of intrinsic and TAM doped $PC_{61}BM$. TAM doping lead to phase separation in $PC_{61}BM$, suggesting that "side-doping" uniform microstructure could mostly form between TAM and long sidechain modified semiconductor.

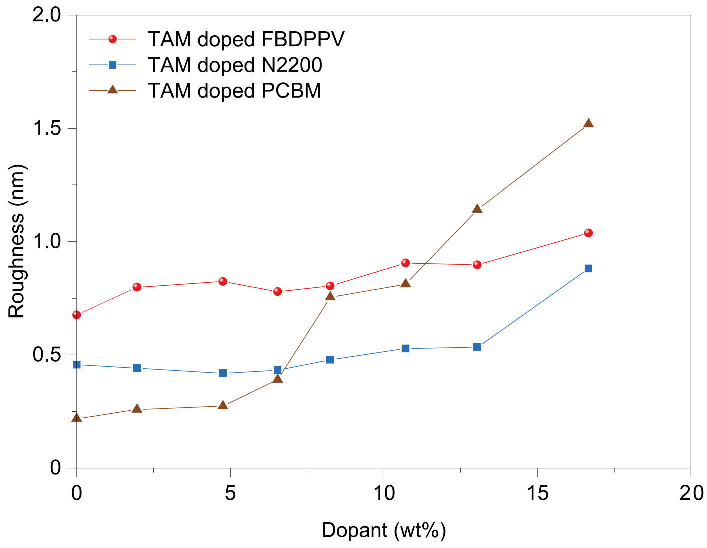

Supplementary Figure 50 | AFM analysis. Surface Roughness of intrinsic and TAM doped FBDPPV, N2200, and $PC_{61}BM$. TAM doped long sidechain modified semiconductor films present smooth surfaces.

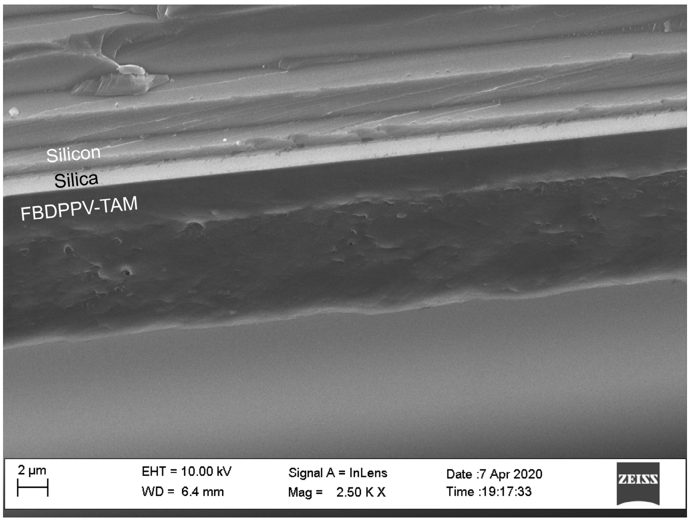

Supplementary Figure 51 | SEM analysis. Scanning electron microscope (SEM) image recorded at 10.00 kV acceleration voltage showing the cross section of TAM doped FBDPPV (5% TAM, at the electrical conductivity maximum, 10 µm) drop-casted on a Si/SiO_2 (1µm SiO_2) substrate. The SEM analysis shows that TAM doped FBDPPV is a compact film.

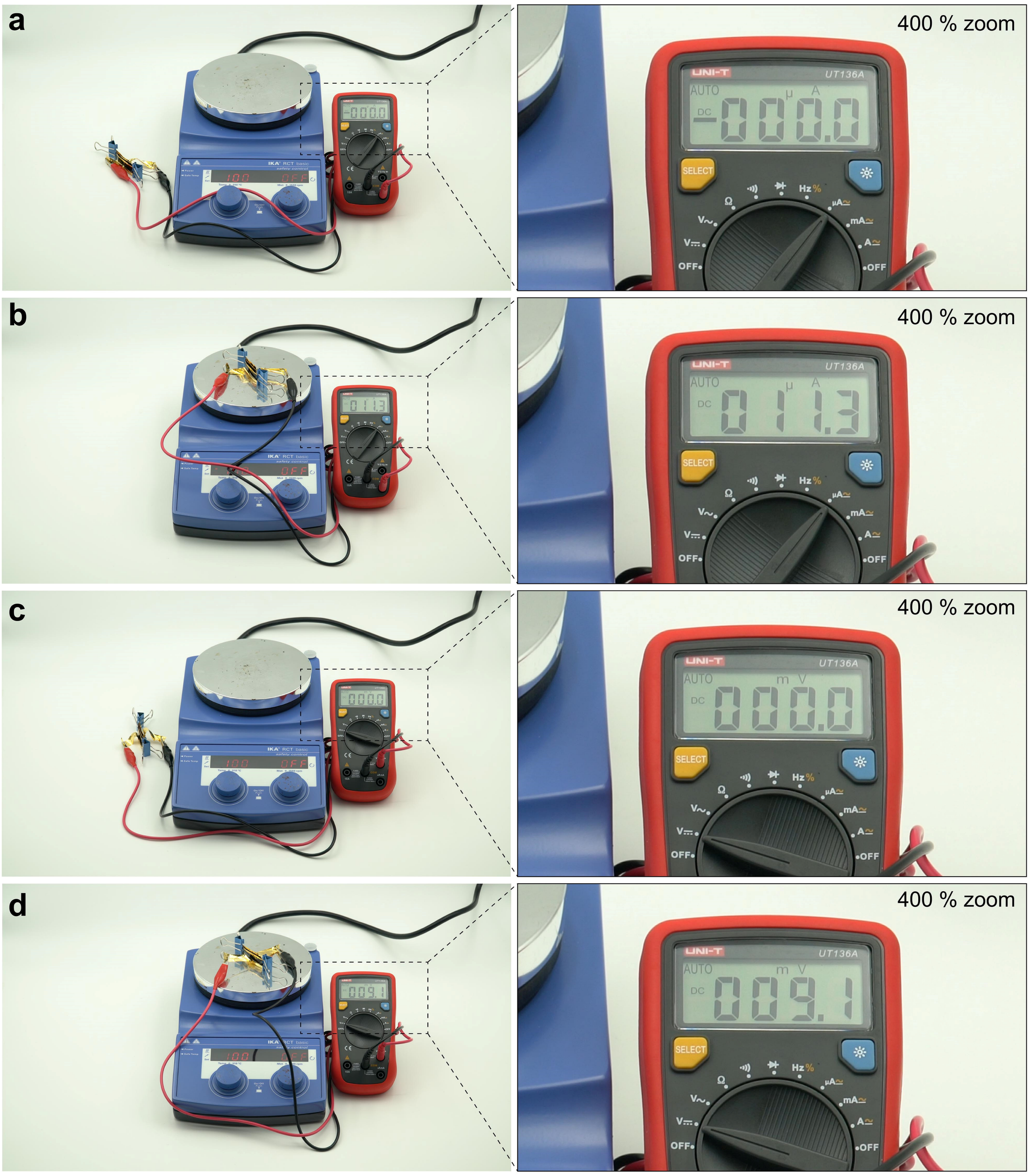

Supplementary Figure 52 | Measurement of thermoelectric generator. a-b Short-circuit current (I_{SC}) measurement under heat source temperature of 100 ºC, air temperature of 25 ºC. **c-d** Open-circuit voltage (V_{OC}) measurement under heat source temperature of 100 ºC, air temperature of 25 ºC. The whole video is available in Supplementary Movie 1.

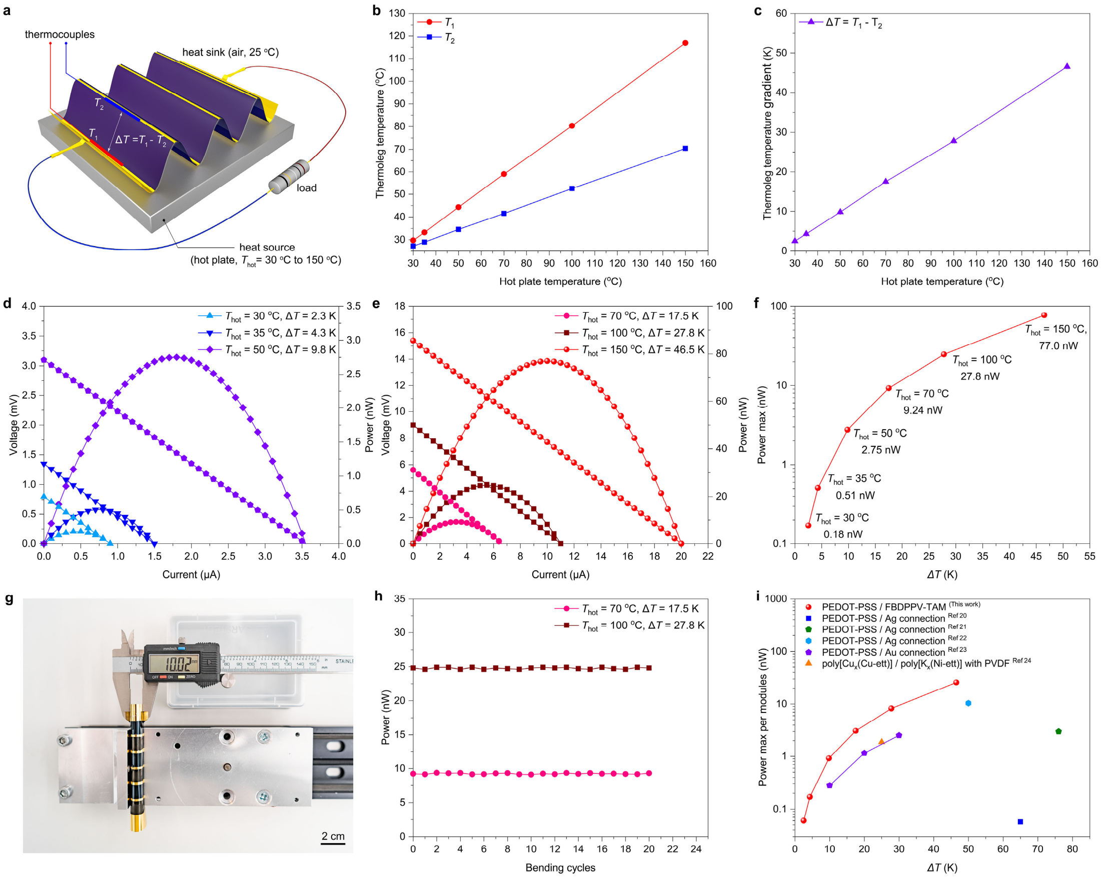

Supplementary Figure 53 | Power output and flexibility of thermoelectric generator. a Measuring method of temperature gradient ($\Delta T = T_1 - T_2$) on thermolegs, T_2 and T_1 are temperatures of the top and end of thermolegs, respectively. T_1 and T_2 are measured using thermocouples which are sticked tightly on thermoleges with kapton tape. **b-c** Temperature gradient on thermolegs with different hot plate temperature. **d-e** Output voltage and power of the generator at different temperature gradient. **f** Maximum power output of the generator at different temperature gradient. **g** Photograph showing bending radius of the legs of thermoelectric generator. **h** Power output of the thermoelectric generator during 20 bending cycles (bending radius = 1 cm). **i** Comparison of maximum power output (per number of thermos modules) of some polymer (including coordination polymer) thermoelectric generators with similar device geometries[20-24]. The all-polymer thermoelectric generator presents good flexibility with high power output.

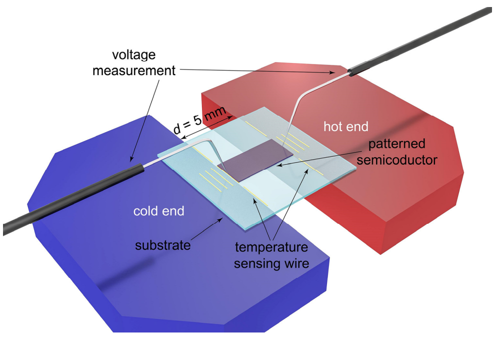

Supplementary Figure 54 | Thermoelectric parameter measurements. Schematic of the geometry of the contacts for electronic conductivity and Seebeck coefficient measurements.

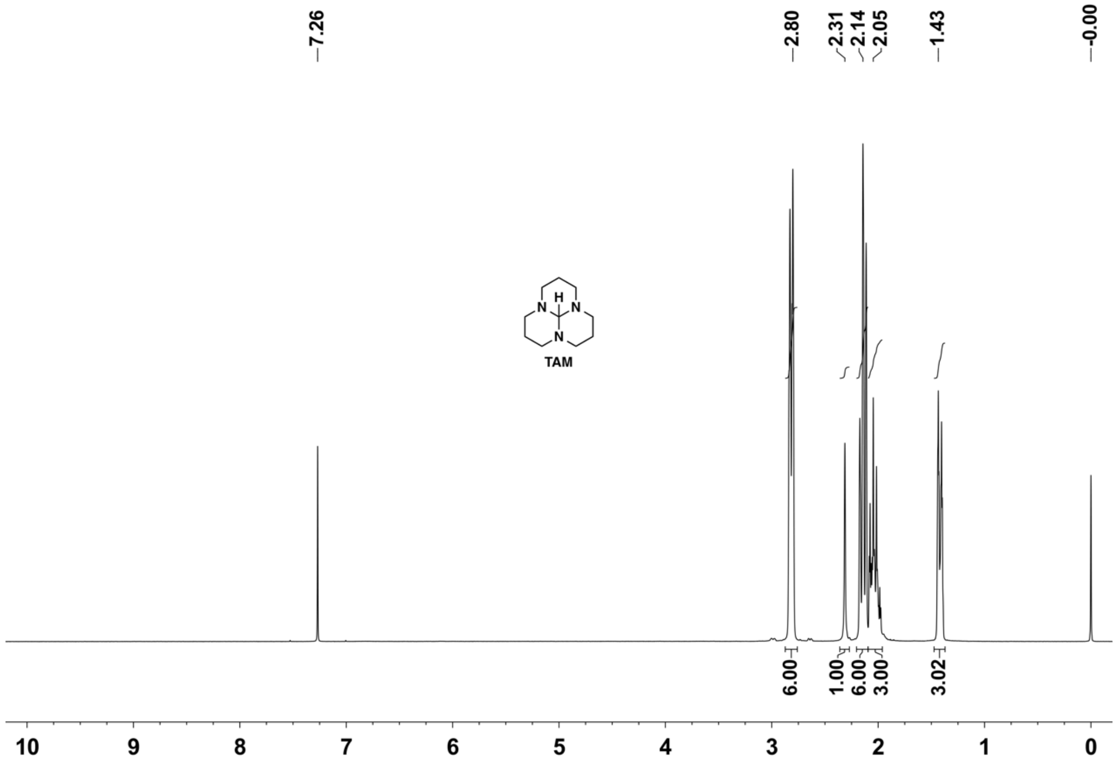

Supplementary Figure 55 | NMR spectrum. ^{1}H-NMR spectrum of TAM in in chloroform-d (298K, 400 MHz).

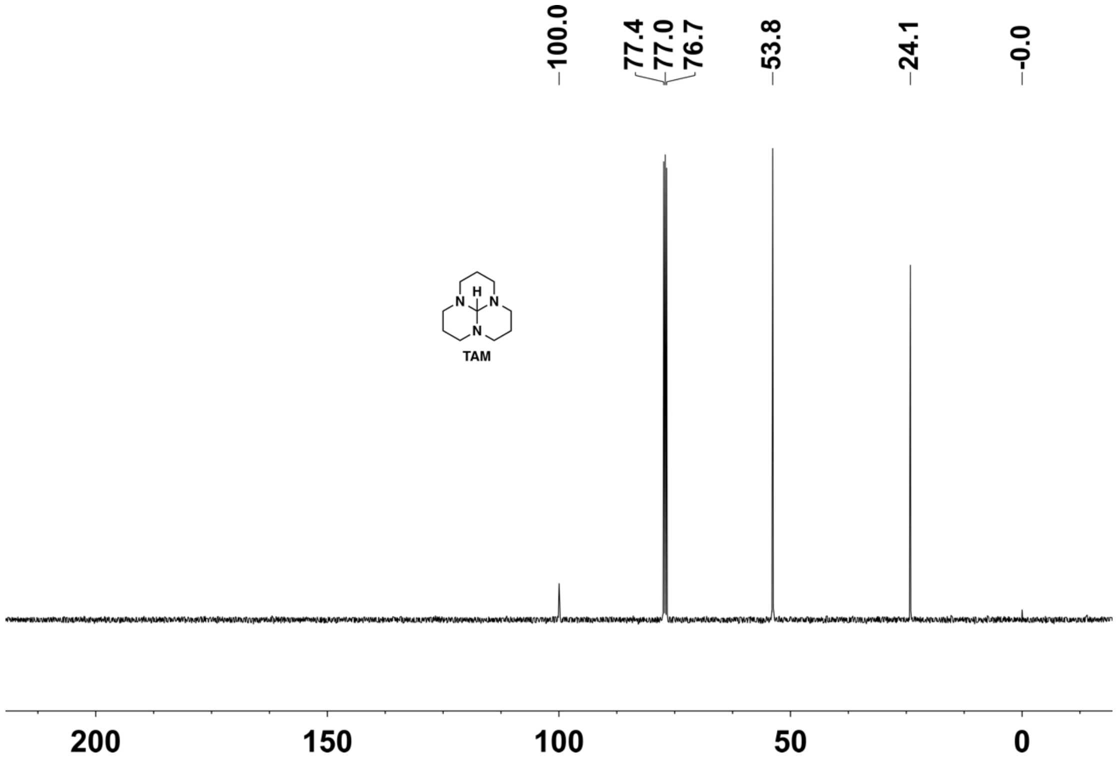

Supplementary Figure 56 | NMR spectrum. ^{13}C-NMR spectrum of TAM in chloroform-d (298K, 101 MHz).

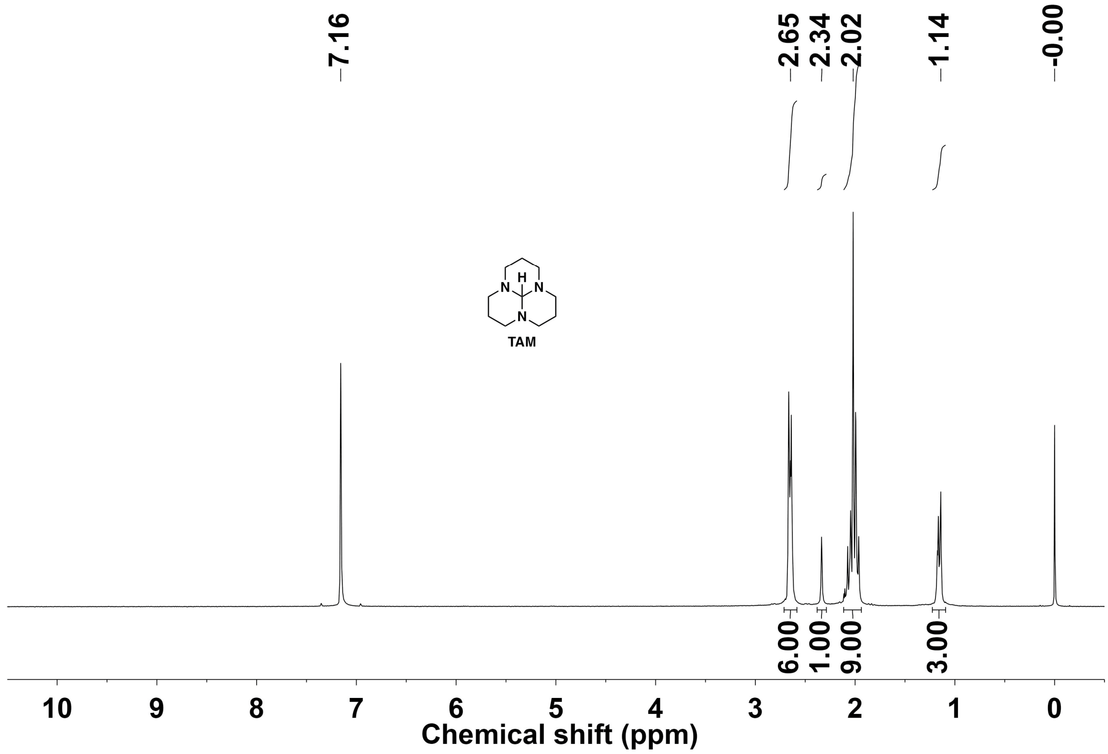

Supplementary Figure 57 | NMR spectrum. ^{1}H-NMR spectrum of TAM in benzene-d_6 (298K, 400 MHz).

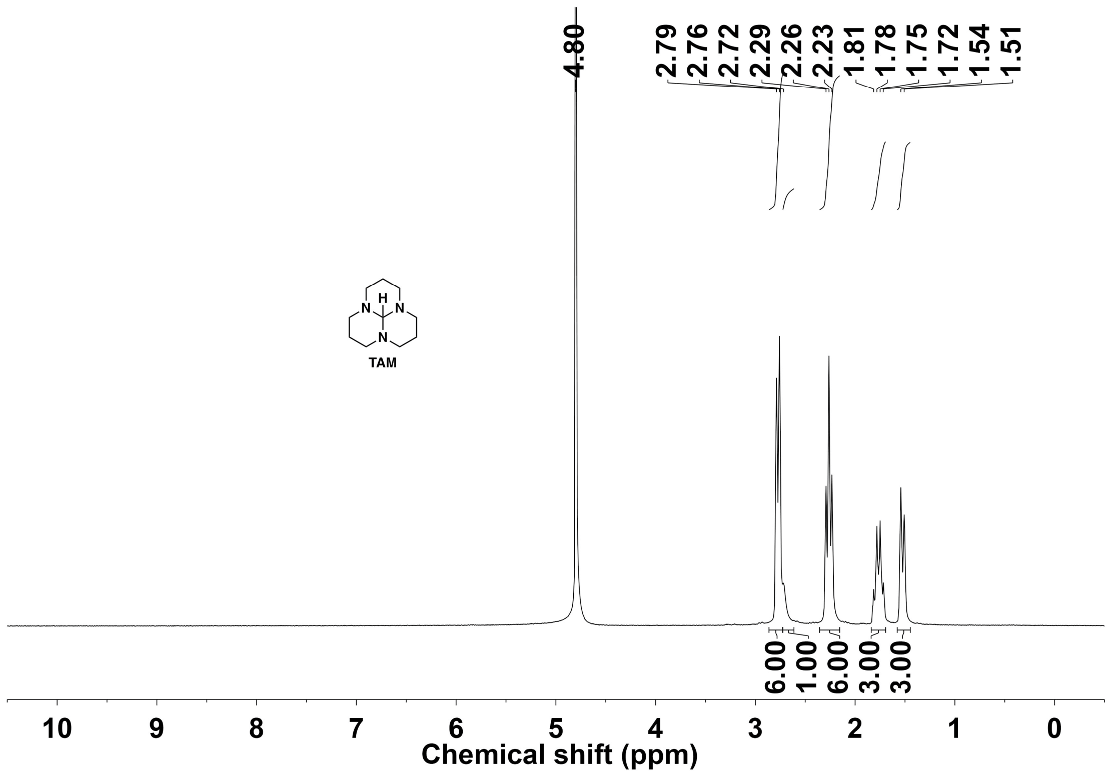

Supplementary Figure 58 | NMR spectrum. ^{1}H-NMR spectrum of TAM in water-d_2 (298K, 400 MHz).

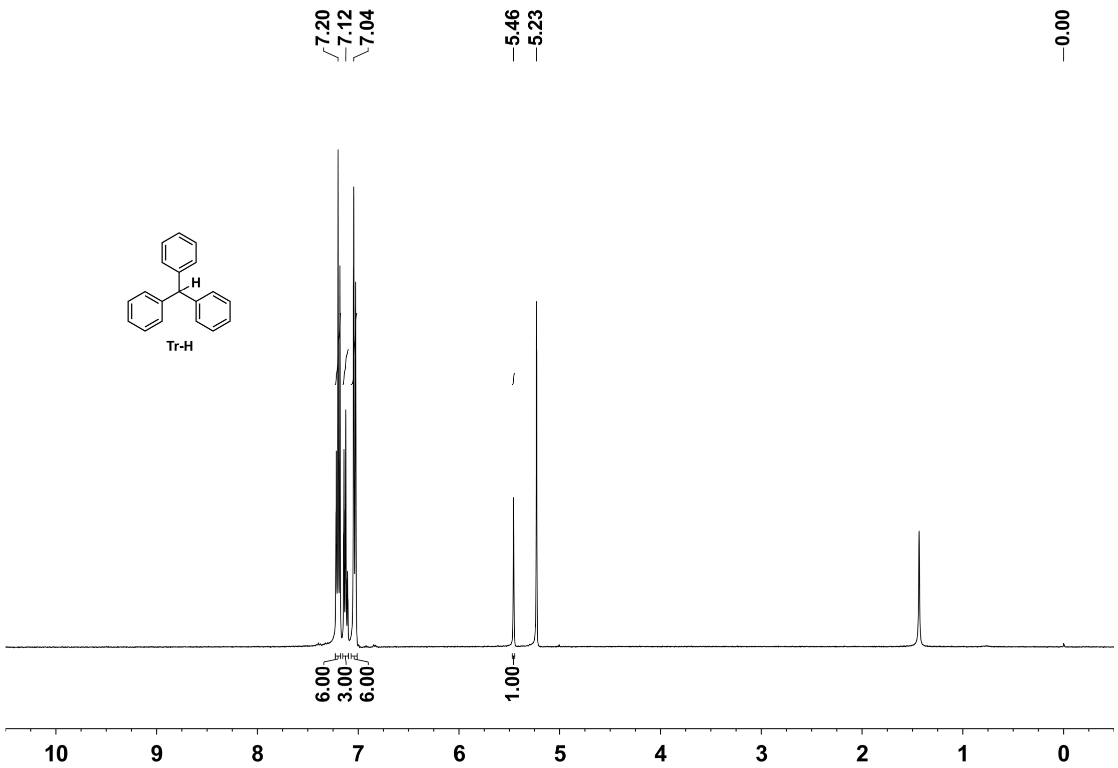

Supplementary Figure 59 | NMR spectrum. ^{1}H-NMR spectrum of triphenylmethane (Tr-H) in dichloromethane-d$_2$ (298K, 400 MHz).

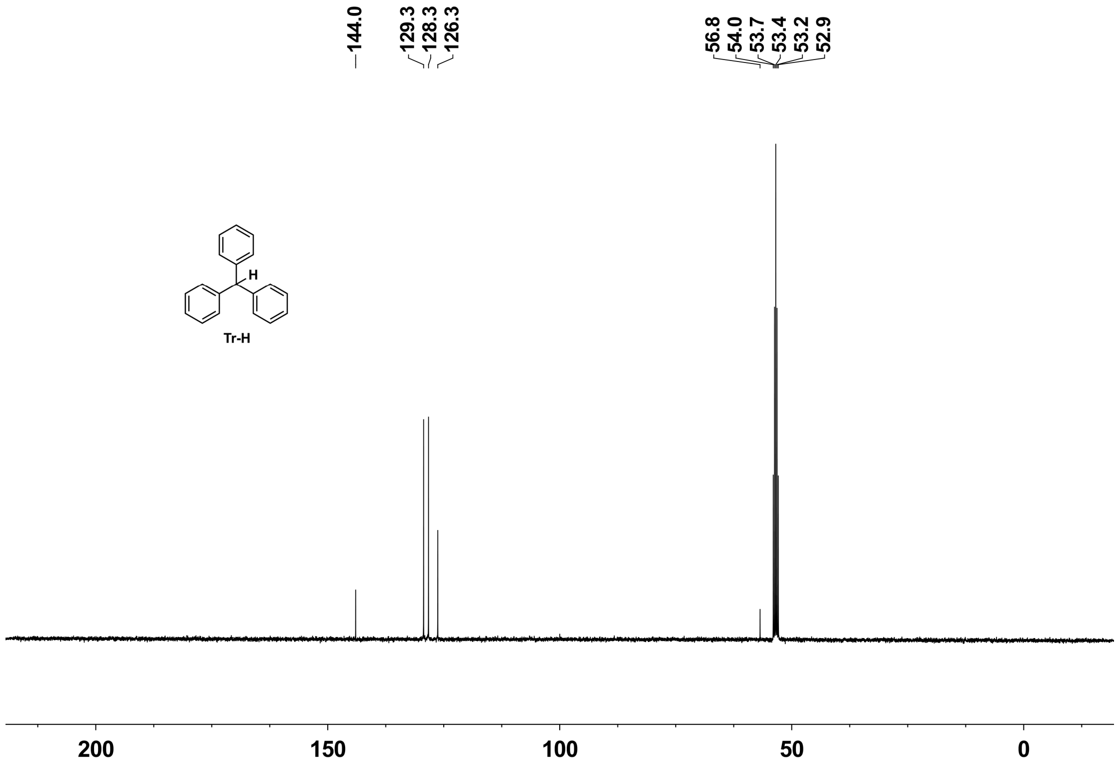

Supplementary Figure 60 | NMR spectrum. ^{13}C-NMR spectrum of triphenylmethane (Tr-H) in dichloromethane-d$_2$ (298K, 101 MHz).

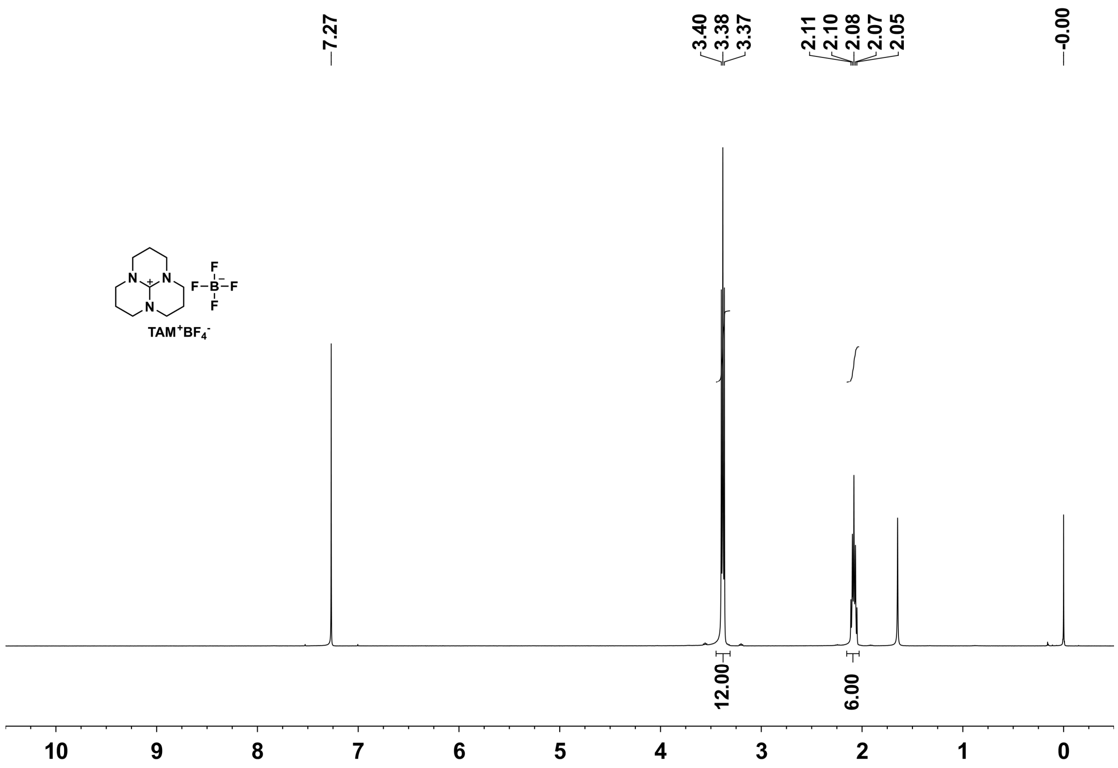

Supplementary Figure 61 | NMR spectrum. ^{1}H-NMR spectrum of TAM^{+} cation (TAM^{+}BF$_4^{-}$) in chloroform-d (298K, 400 MHz).

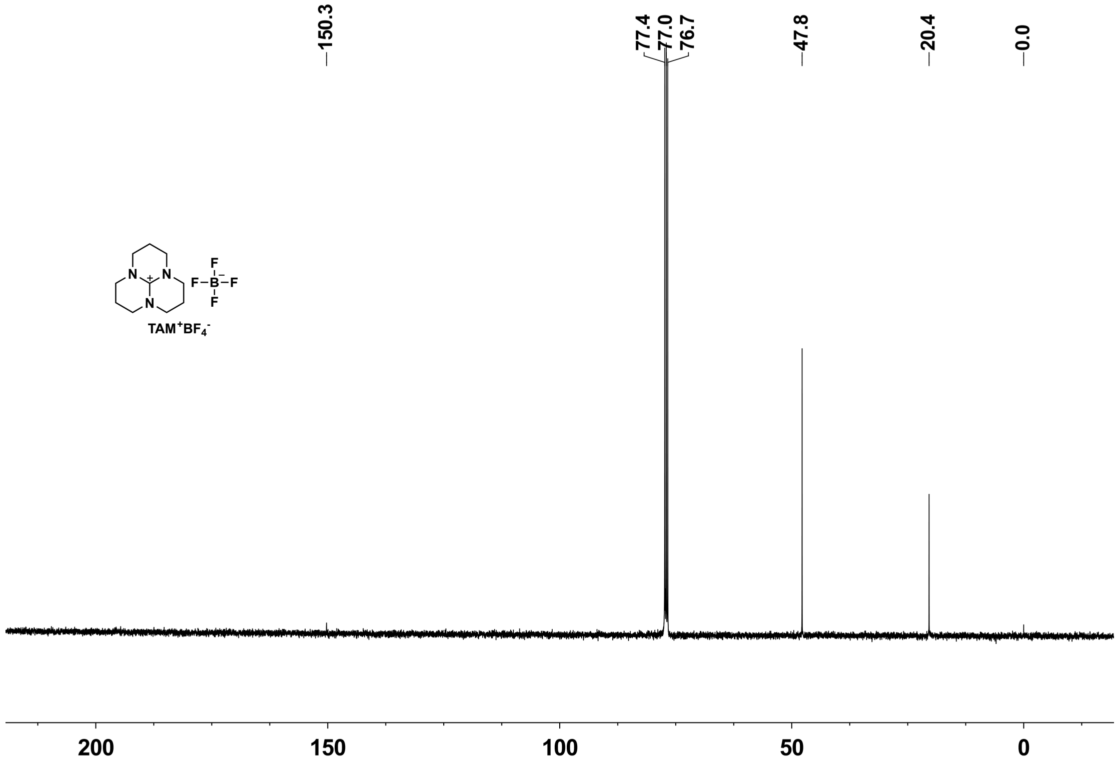

Supplementary Figure 62 | NMR spectrum. ^{13}C-NMR spectrum of TAM^{+} cation (TAM^{+}BF$_4^{-}$) in chloroform-d (298K, 101 MHz).

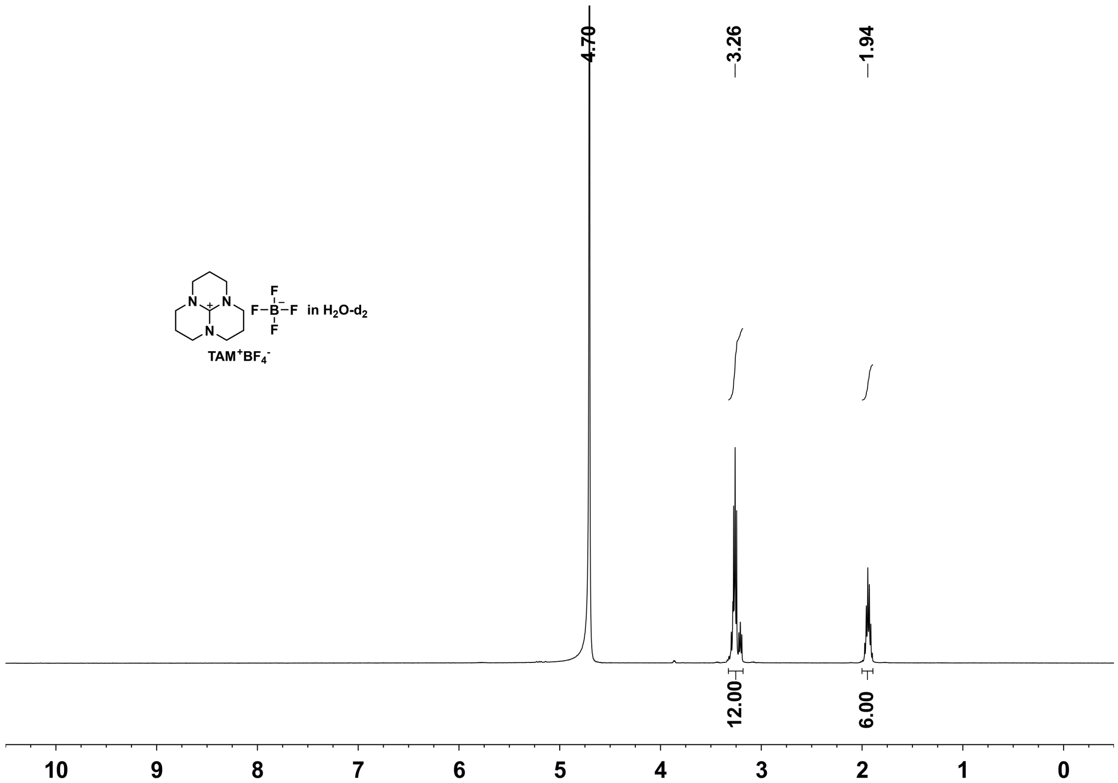

Supplementary Figure 63 | NMR spectrum. ^{1}H-NMR spectrum of TAM$^+$ cation (TAM$^+$BF$_4^-$) in water-d$_2$ (298K, 400 MHz).

.

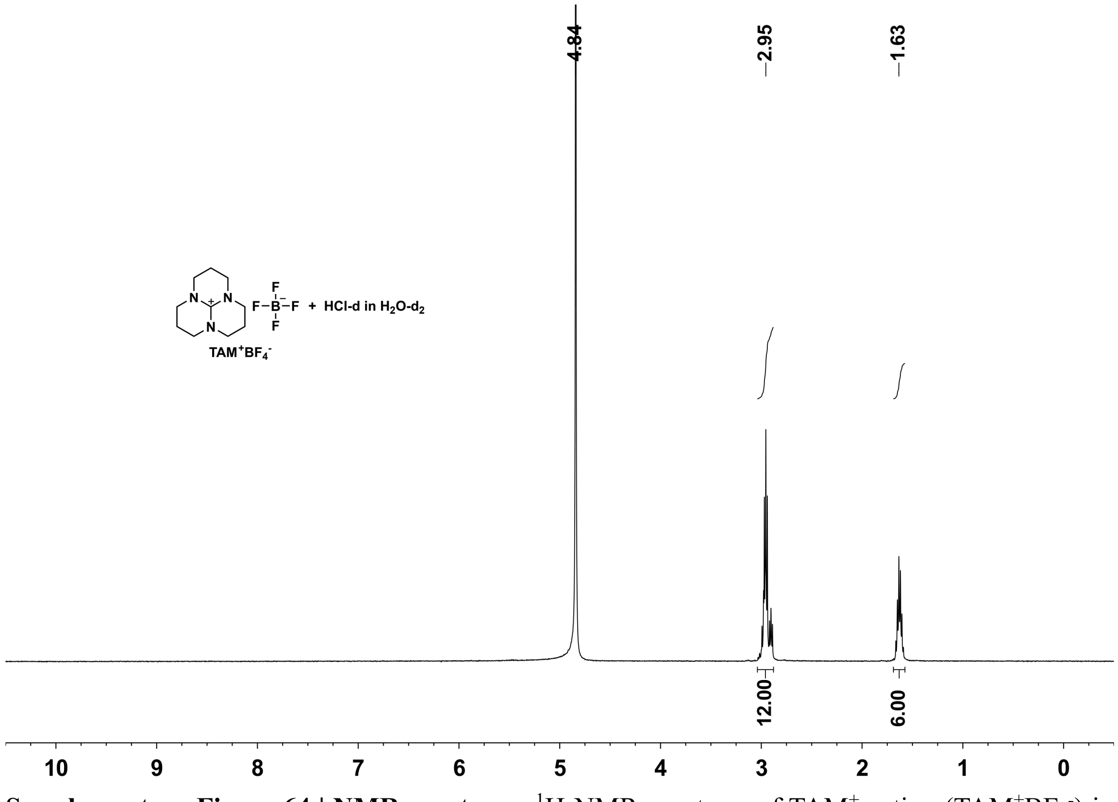

Supplementary Figure 64 | NMR spectrum. ^{1}H-NMR spectrum of TAM$^+$ cation (TAM$^+$BF$_4^-$) in saturated HCl-d (H_2O-d$_2$ solution, 298K, 400 MHz)

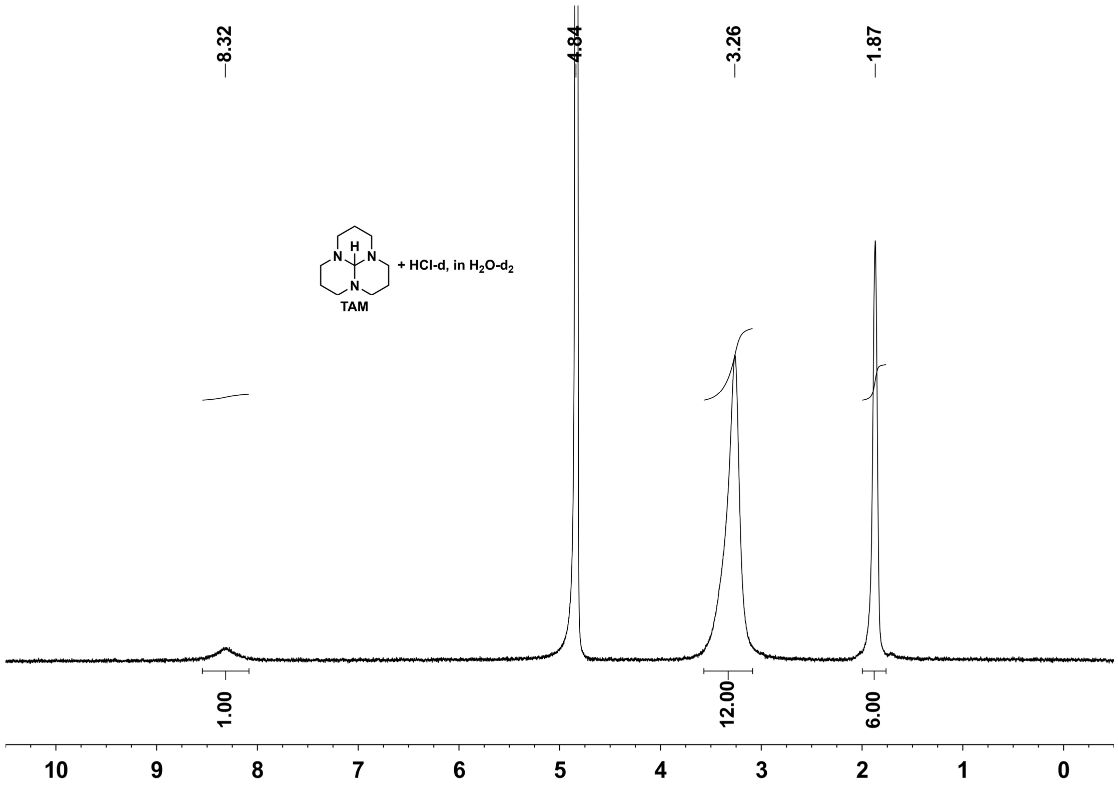

Supplementary Figure 65 | NMR spectrum. ^{1}H-NMR spectrum of TAM in saturated HCl-d (H_2O-d_2 solution, 298K, 400 MHz)

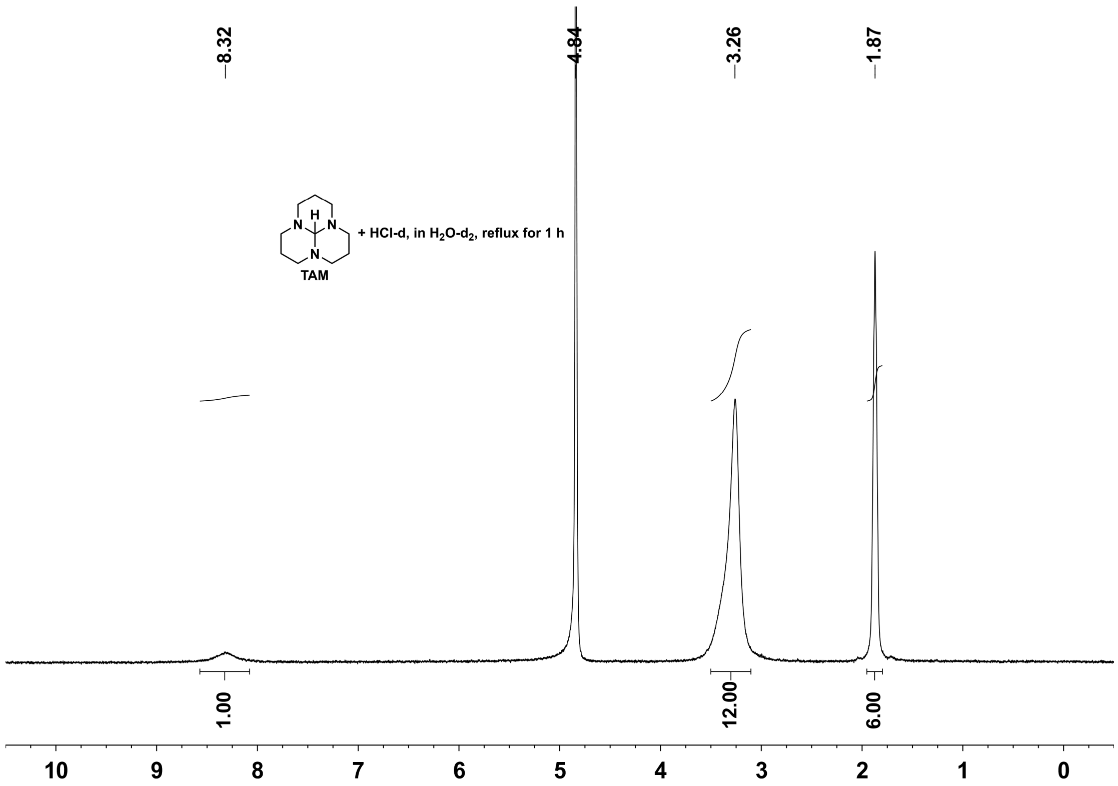

Supplementary Figure 66 | NMR spectrum. ^{1}H-NMR spectrum of TAM in saturated HCl-d (H_2O-d_2 solution, after boiling for 1 hour, 298K, 400 MHz)

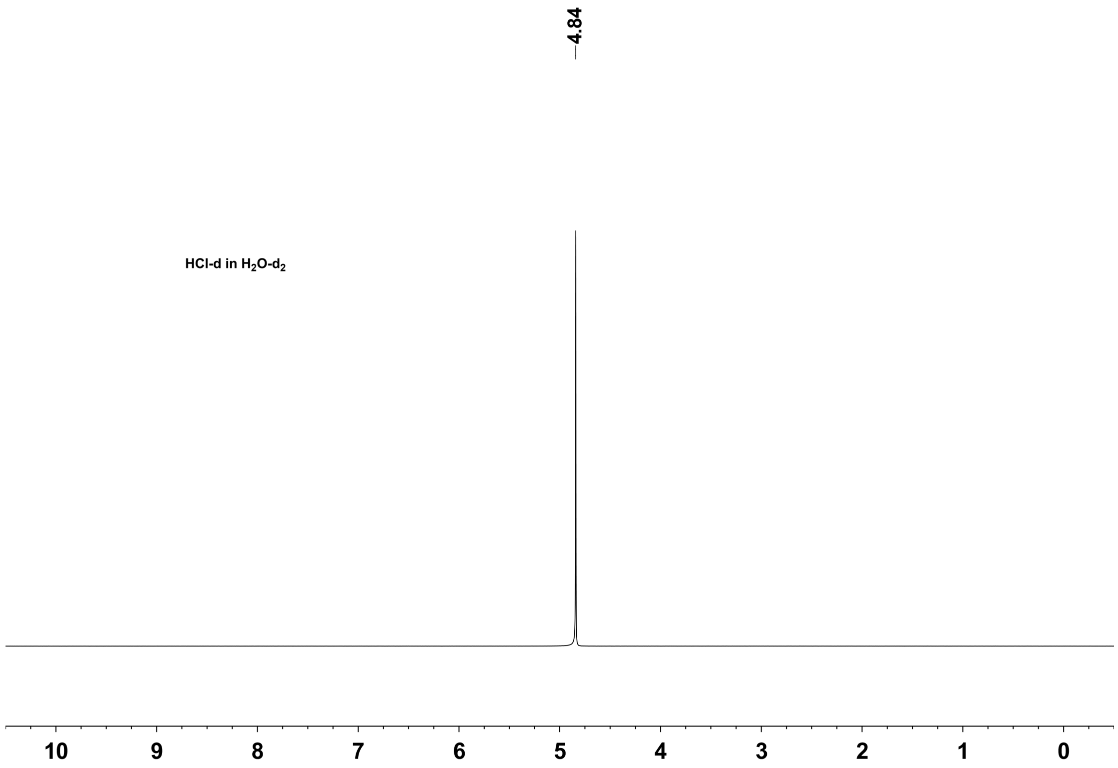

Supplementary Figure 67 | NMR spectrum. ^{1}H-NMR spectrum of saturated HCl-d in H_2O-d_2 (298K, 400 MHz)

Supplementary Tables

Supplementary Table 1 | DFT calculations on n-doping ability prediction [a].

Compounds	ΔG_{H^-} (eV)	$\Delta G_{H\cdot}$ (eV)	SOMO Level (eV)	$d_{C\text{–}H}$ (Å)	$\delta^1{}_{H\text{-NMR}}$ (ppm)	Mulliken Charge (a.u.)
TAM_{Hy}	7.75	3.59	-3.62	1.095	4.31	0.145
TAM_{Me}	7.02	3.33	-3.16	1.102	3.05	0.106
TAM_{Et}	6.80	3.36	-3.20	1.097	4.10	0.108
TAM_{Pr}	6.77	3.67	-3.09	1.086	4.53	0.122
TAM_{3T}	8.06	3.62	-3.91	1.124	0.47	0.040
TAM_{3Q}	6.93	3.43	-3.14	1.097	2.72	0.135
TAM_{3P}	6.79	3.40	-2.80	1.092	4.13	0.139
TAM_{3H}	6.82	3.29	-3.06	1.088	3.19	0.102
TAM_{555}	7.91	3.82	-3.84	1.093	5.03	0.184
TAM_{556}	6.85	3.53	-3.01	1.120	4.04	0.106
TAM_{566}	6.70	3.43	-2.97	1.135	2.49	0.101
TAM	6.82	3.46	-3.08	1.130	2.31	0.116
TAM_{667}	6.77	3.41	-3.00	1.126	2.82	0.102
TAM_{677}	6.92	3.55	-3.14	1.117	2.95	0.103
TAM_{777}	7.02	3.68	-3.29	1.113	3.50	0.110
TAM_{Me2T}	7.87	3.78	-3.55	1.112	1.77	0.071
TAM_{Me5}	7.35	3.62	-3.41	1.109	4.12	0.105
TAM_{Me6}	7.26	3.55	-3.50	1.116	2.63	0.090
TAM_{Me66}	6.71	3.38	-2.99	1.124	2.90	0.091

[a] Under B3LYP/6-311+G(d,p) level.

Supplementary Table 2 | DFT calculations on n-doping ability prediction [a].

Compounds	ΔG_{H^-} (eV)	$\Delta G_{H\cdot}$ (eV)	SOMO Level (eV)	$d_{C\text{–}H}$ (Å)	$\delta^1{}_{H\text{-NMR}}$ (ppm)	Mulliken Charge (a.u.)
TAM	6.82	3.46	-3.08	1.130	2.31	0.116
N-DMBI	6.97	3.24	-2.72	1.116	4.78	0.088
LCV	7.37	3.05	-3.48	1.097	4.39	0.119
LPB	7.67	3.01	-3.51	1.097	5.30	0.176
DPDHP	6.92	2.47	-3.67	1.102	3.76	0.132

[a] Under B3LYP/6-311+G(d,p) level.

Supplementary Table 3 | DFT calculated molecular polarizability[a].

Compounds	Dipole moment (Debye)	Polarizability (a.u.)	Molecular mass (dalton)	Total electrons	Valence electrons	Normalized polarizability[b] (a.u.)
1. TAM_{Hy}^+	0.00	32.43	60.08	32	24	1.35
2. TAM_{Me}^+	0.00	106.83	144.24	80	60	1.78
3. TAM_{Et}^+	0.00	178.66	228.40	129	97	1.84
4. TAM_{Pr}^+	0.00	253.94	312.34	176	132	1.92
5. TAM_{3T}^+	2.21	98.26	138.19	74	54	1.82
6. TAM_{3Q}^+	0.01	130.98	180.15	98	72	1.82
7. TAM_{3P}^+	0.00	166.77	222.36	122	90	1.85
8. TAM_{3H}^+	0.82	202.79	264.24	146	108	1.88
9. TAM_{555}^+	1.93	89.26	138.19	74	54	1.65
10. TAM_{556}^+	0.30	102.57	152.22	82	60	1.71
11. TAM_{566}^+	0.45	114.33	166.25	90	66	1.73
12. TAM^+	0.46	126.29	180.28	98	72	1.75
13. TAM_{667}^+	0.99	137.30	194.30	106	78	1.76
14. TAM_{677}^+	0.91	148.66	208.33	114	84	1.77
15. TAM_{777}^+	0.22	160.23	222.36	122	90	1.78
16. TAM_{Me2T}^+	1.50	99.04	140.21	76	56	1.77
17. TAM_{Me5}^+	0.22	100.81	142.23	78	58	1.74
18. TAM_{Me6}^+	0.14	112.87	156.15	86	64	1.76
19. TAM_{Me66}^+	0.16	119.21	168.15	92	68	1.75
20. $N\text{-}DMBI^+$	0.27	247.69	266.37	142	102	2.43
21. $DPDHP^+$	2.29	342.28	352.46	186	132	2.59
22. LCV^+	0.01	460.89	372.54	200	144	3.20
23. LPB^+	0.01	357.03	323.46	174	126	2.83
24. $n\text{-}C_{10}H_{22}$	0.00	124.41	142.29	82	62	2.01
25. $n\text{-}C_{14}H_{30}$	0.00	174.21	198.39	114	86	2.03
26. $n\text{-}C_{18}H_{38}$	0.00	224.22	254.50	146	110	2.04
27. $n\text{-}C_{22}H_{46}$	0.00	274.28	310.61	178	134	2.05
28. $n\text{-}C_{26}H_{54}$	0.00	324.41	366.72	210	158	2.05
29. $n\text{-}C_{30}H_{62}$	0.00	374.53	422.83	242	182	2.06
30. naphthalene	0.00	116.57	128.17	68	48	2.43
31. anthracene	0.00	178.26	178.23	94	66	2.70
32. tetracene	0.00	251.41	228.29	120	84	2.99
33. pentacene	0.00	335.34	278.35	146	102	3.29
34. N2200-1	2.44	422.65	458.51	236	156	2.71
35. FBDPPV-1	1.06	596.28	538.46	276	196	3.04

[a] Under B3LYP/6-311+G(d,p) level. [b] Normalized by number of valence electrons.

Supplementary Table 4 | TD-DFT calculations on absorption spectra. TD-DFT calculated vertical excitation energy (ΔE_{ve}), corresponding wavelength and oscillator strength of FBDPPV trimer under B3LYP/6-31G(d) level.

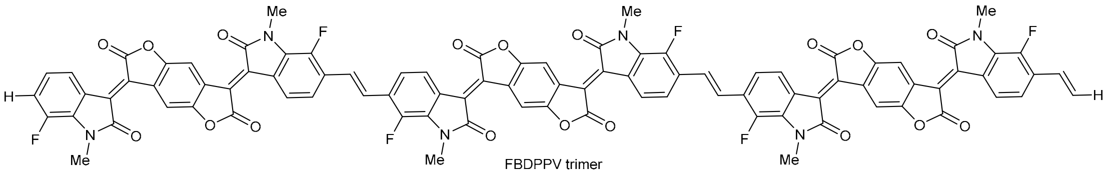

FBDPPV trimer

Excited states	ΔE_{ve} (eV)	Wavelength (nm)	Oscillator strengths	Excited states	ΔE_{ve} (eV)	Wavelength (nm)	Oscillator strengths
**S1**	**1.5046**	**824.13**	**4.2760**	S26	2.5388	488.42	0.1311
S2	1.7537	707.07	0.0182	S27	2.5430	487.62	0.0007
S3	1.7784	697.27	0.0066	S28	2.5575	484.84	0.0035
S4	1.9061	650.55	0.0023	S29	2.5613	484.13	0.0379
**S5**	**1.9369**	**640.21**	**0.3452**	S30	2.5866	479.39	0.0018
S6	1.9548	634.33	0.0906	S31	2.5901	478.74	0.0001
S7	1.9894	623.29	0.0007	S32	2.6239	472.58	0.0449
S8	2.0350	609.34	0.0074	S33	2.6417	469.39	0.0132
S9	2.0452	606.30	0.0006	S34	2.6647	465.34	0.0530
S10	2.0922	592.69	0.0012	S35	2.6962	459.91	0.0010
S11	2.1342	581.02	0.1611	S36	2.7022	458.89	0.0011
S12	2.1455	577.96	0.0394	S37	2.7096	457.64	0.0037
S13	2.1627	573.37	0.0896	S38	2.7199	455.90	0.0048
S14	2.1707	571.24	0.0280	S39	2.7376	452.95	0.0137
S15	2.2608	548.47	0.0203	S40	2.7705	447.57	0.0013
**S16**	**2.2703**	**546.19**	**0.2928**	S41	2.7831	445.54	0.0356
S17	2.2958	540.12	0.0415	S42	2.8071	441.74	0.0306
**S18**	**2.3377**	**530.44**	**0.4873**	S43	2.8165	440.27	0.0028
S19	2.3561	526.29	0.0025	S44	2.8544	434.41	0.0531
S20	2.3913	518.54	0.0681	S45	2.8790	430.70	0.0168
S21	2.4163	513.18	0.0205	S46	2.8884	429.31	0.0331
S22	2.4605	503.96	0.0005	S47	2.8933	428.58	0.0009
S23	2.4629	503.47	0.0201	S48	2.9217	424.41	0.0232
S24	2.4712	501.79	0.0137	S49	2.9419	421.50	0.0167
S25	2.4809	499.81	0.1169	S50	2.9495	420.41	0.0069

Supplementary Table 5 | TD-DFT calculations on absorption spectra. TD-DFT calculated vertical excitation energy (ΔE_{ve}), corresponding wavelength and oscillator strength of (FBDPPV trimer)$^{-}$ anion under B3LYP/6-31G(d) level.

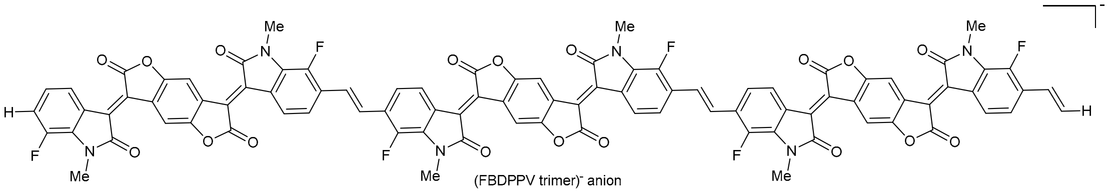

(FBDPPV trimer)$^{-}$ anion

Excited states	ΔE_{ve} (eV)	Wavelength (nm)	Oscillator strengths
**D1**	**0.3575**	**3468.55**	**1.8182**
D2	0.5077	2442.37	0.0001
D3	0.9186	1349.88	0.0135
D4	0.9564	1296.56	0.0439
**D5**	**0.9927**	**1249.14**	**1.1161**
D6	1.0312	1202.53	0.0004
D7	1.0442	1187.52	0.0112
D8	1.1951	1037.57	0.1039
D9	1.3509	917.90	0.0083
**D10**	**1.4558**	**851.78**	**1.2235**
D11	1.4854	834.81	0.0224
**D12**	**1.5294**	**810.76**	**0.4484**
D13	1.5604	794.68	0.0106
D14	1.6081	771.08	0.0120
**D15**	**1.6942**	**731.93**	**0.4796**
D16	1.7853	694.57	0.0005
D17	1.8090	685.45	0.0817
D18	1.8478	671.08	0.0020
D19	1.8639	665.28	0.0074
D20	1.8727	662.15	0.0557
D21	1.9214	645.36	0.0013
D22	1.9267	643.58	0.0002
D23	1.9335	641.33	0.0000
D24	1.9526	635.06	0.0568
D25	1.9543	634.51	0.0010
D26	1.9692	629.71	0.0250
D27	2.0050	618.44	0.0002
D28	2.0184	614.35	0.0172
D29	2.0325	610.09	0.0034
D30	2.0483	605.39	0.0530
D31	2.0678	599.68	0.0156
D32	2.0752	597.53	0.0555
D33	2.0937	592.24	0.0109
D34	2.1013	590.11	0.0008
D35	2.1198	584.97	0.0003
D36	2.1374	580.14	0.0491
D37	2.1593	574.26	0.0509
D38	2.1684	571.86	0.0012
D39	2.1779	569.36	0.0313
D40	2.1920	565.69	0.0009
D41	2.1976	564.24	0.0049
D42	2.2116	560.69	0.0029
D43	2.2318	555.60	0.0010
D44	2.2422	553.04	0.0345
D45	2.2631	547.93	0.0230
D46	2.2748	545.10	0.0251
D47	2.2785	544.21	0.0124
D48	2.2974	539.75	0.0056
D49	2.3129	536.13	0.0049
D50	2.3155	535.53	0.1460

Supplementary Table 6 | TD-DFT calculations on absorption spectra. TD-DFT calculated vertical excitation energy (ΔE_{ve}), corresponding wavelength and oscillator strength of (FBDPPV trimer)$^{2-}$ dianion under B3LYP/6-31G(d) level.

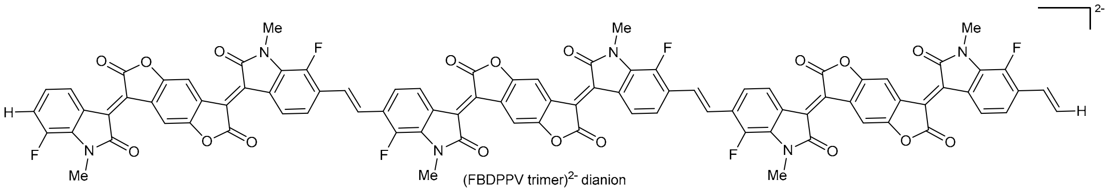

Excited states	ΔE_{ve} (eV)	Wavelength (nm)	Oscillator strengths
**S1**	**0.5858**	**2116.62**	**4.7046**
S2	0.7404	1674.75	0.0000
S3	1.0686	1160.40	0.0121
S4	1.2100	1024.83	0.0001
S5	1.3459	921.35	0.0020
S6	1.3891	892.65	0.0433
**S7**	**1.4951**	**829.40**	**1.3919**
S8	1.7469	709.84	0.0014
S9	1.7618	703.83	0.1082
S10	1.8433	672.69	0.0001
S11	1.9545	634.43	0.0462
S12	2.0131	615.96	0.0086
S13	2.0369	608.77	0.0136
S14	2.0530	604.00	0.1144
S15	2.1022	589.85	0.0001
S16	2.1214	584.52	0.0001
S17	2.1477	577.36	0.0063
S18	2.1609	573.84	0.0093
S19	2.1754	570.01	0.0071
S20	2.1944	565.08	0.0096
S21	2.2286	556.40	0.0226
S22	2.2331	555.27	0.0051
S23	2.2502	551.07	0.1022
S24	2.2895	541.61	0.0013
S25	2.3543	526.69	0.1289

Excited states	ΔE_{ve} (eV)	Wavelength (nm)	Oscillator strengths
S26	2.3881	519.25	0.0481
S27	2.4049	515.62	0.0758
S28	2.4422	507.74	0.0469
S29	2.4569	504.71	0.0153
S30	2.4780	500.41	0.0452
S31	2.5003	495.94	0.0015
S32	2.5303	490.06	0.0232
S33	2.5601	484.35	0.0004
S34	2.5811	480.41	0.0017
S35	2.6195	473.38	0.2054
S36	2.6234	472.67	0.0006
S37	2.6398	469.73	0.0160
S38	2.6681	464.75	0.0122
S39	2.6754	463.48	0.0159
S40	2.7093	457.68	0.0005
S41	2.7783	446.32	0.0097
S42	2.7830	445.56	0.0162
S43	2.7975	443.26	0.0033
S44	2.8116	441.03	0.0404
S45	2.8359	437.25	0.0026
S46	2.8602	433.54	0.0034
S47	2.8967	428.08	0.0002
S48	2.9137	425.58	0.0172
S49	2.9455	420.98	0.0004
S50	2.9533	419.87	0.0035

Supplementary Notes

Supplementary Note 1 | Design of TAMs and n-doping ability prediction. The major structures of TAMs were conceived of by intuitive design and then evaluated. The intuitive design followed simple principle: 1) choosing triaminomethane building block, because it can form a stable guanidine cation after reduction. This comes from our long-time molecular design intuitive and literature research; 2) introducing alkyls to nitrogen atoms to enhance the electron-donating property of TAMs; 3) investigating the effect of methyl and longer alkyl substitutions and evaluate the redox properties and stabilities of the compound by calculation; 4) reducing steric hindrance by inspecting cyclic/fused cyclic alkyl substitutions; 5) inspecting more possible combinations of liner and cyclic alkyl substitutions

Supplementary Figure 2 (a) illustrates that compared with hydrogens, methyl substitutions on TAMs could obviously enhance the predicted n-doping ability. This can be attributed to the stronger electron-donating property of alkyl than that of hydrogen. Further increasing the alkyl length to ethyl or propyl would lead to decreased predicted n-doping ability. This suggests that ethyl or propyl does not showing obviously stronger electron-donating property than methyl, and while the larger steric hindrances would affect their planarity thus affect their predicted n-doping abilities.

Intuitively, cyclic alkyls would probably show smaller steric hindrances. The predicted n-doping abilities of TAMs with three to six membered aza cyclic alkyls are shown in Supplementary Figure 2 (b). TAM_{3T} shows unbalanced predicted n-doping ability, indicating that although aziridine is an electron-rich donor but its tension would be unfavorable to stabilize the cation. TAM_{3Q} shows better balanced predicted n-doping ability which is similar to TAM_{Me}, and further increasing ring size to TAM_{3P}/TAM_{3H} would lead to decreased predicted n-doping ability. This suggests that inter-ring steric hindrance in TAM_{3P} and TAM_{3H} may reduce n-doping ability.

Therefore, fused alkyl ring substituted TAMs are inspected to reduce such inter-ring steric hindrance. Supplementary Figure 2(c) demonstrates that TAM_{566}, TAM and TAM_{667} could have the predicted strongest n-doping ability among all TAMs, and either reducing or increasing ring size may lead to decreased n-doping ability. This indicates that fused aza six-membered rings would be favored to enhance the n-doping ability due to their specific chair conformations and secondary orbital interactions (Supplementary Figure 3).

Furthermore, some combinations of rings and methyl substituted TAMs were also investigated. Supplementary Figure 2 (d) shows that among these combinations, only TAM_{Me66} could have strong n-doping ability comparable to TAM_{566}, TAM and TAM_{667}. This implies that only the fused aza six-membered rings (1,5,7-triazabicyclo[4.4.0]decane, Supplementary Figure 2(e)) backbone might be essential for high predicted n-doping ability in TAM derivatives.

Supplementary Note 2 | Molecular polarizability and cation-side chain interaction. Since both TAM^+ and $N\text{-}DMBI^+$ have very small dipole moments and alkyl sidechains are almost nonpolar, only dispersion and induction forces are important in cation-alkylchain interactions. Molecular polarizability is one of the most important part in dispersion and induction forces[1]. As polarizability relates to molecular volume, so it is hard to direct compare the polarizability property of different size of molecules. DFT calculations shows that in *n*-alkanes, molecular polarizabilities are almost proportional to their number of valence electrons, or number of total electrons, or molecular mass. Assume that *n*-alkanes have the similar polarizability property, thus their polarizability property can

be described by the normalized polarizability (normalized by the number of valence electrons).

DFT calculated normalized polarizability of $TAMs^+$ cations, cations of reported hydride dopants, benzene derivatives (including arenes and monomer of N2200 and FBDPPV backbones), and *n*-alkanes are shown in Supplementary Table2 and Supplementary Figure 10. Supplementary Figure 10 shows that without alkyl substitutions, TAM_{Hy} cation shows smallest normalized polarizability, which is significantly different from other alkylated $TAMs^+$ cations. It is also quite clear that all alkylated $TAMs^+$ cations have small normalized polarizability similar to *n*-alkanes, while N-$DMBI^+$, LCV^+, LPB^+, and $DPDHP^+$ have larger normalized polarizability similar to arenes and polymer backbones. These results suggest that TAM^+ cation has intrinsic strong affinity with alkyl sidechain while N-$DMBI^+$ cation has very weak affinity with alkyl sidechain but strong affinity with polymer backbone due to their matched Van der Waals interactions.

Supplementary Note 3 |Synthesis of TAM+ cation. To a 500 mL two-neck bottle equipped with calcium chloride drying tube, 4.80 g of sodium hydride (60% in naphtha, 120 mmol) was added and washed by hexane. A solution of HHPP (16.7 g, 120 mmol) in 250 mL of anhydrous THF was added to the washed sodium hydride, and 24.2 g of 1,3-dibromopropane (120 mmol) was added dropwise. Then the mixture was stirred for 24 h at room temperature. During the stirring, a large amount of white precipitates were produced. The white precipitates were collected through vacuum filtration, washed with anhydrous ether, and then added to sodium tetrafluoroborate saturated aqueous solution (52.7 g of $NaBF_4$, 480 mmol). The resulting aqueous solution was extracted with dichloromethane, then the organic phase was combined and dried with anhydrous sodium sulfate. After removing all solvents by vacuum distillation, crude TAM^+BF^{4-} were obtained as colorless solids. Then crude were washed with anhydrous tetrahydrofuran and hexane, and dried in vacuum to obtain pure TAM^+BF^{4-} as colorless crystals (27.3 g, 85% yield, Supplementary Figure 14). ^1H NMR ($CDCl_3$, 400 MHz, ppm): δ 3.38 (m, 12H), 2.08 (m, 6H). ^{13}C NMR ($CDCl_3$, 101 MHz, ppm): δ 150.3, 47.8, 20.4. ESI HRMS calcd. for M^+: 180.1495; Found: 180.1496.

Supplementary Note 4 | Hydride-transfer reactions between TAM or N-DMBI and Tr^+. Tritylium tetrafluorobate (Tr^+, 364 mg, 1.10 mmol) was dissolved into 5 mL of anhydrous dichloromethane, and mixed with TAM (200 mg, 1.10 mmol) in 5 mL of anhydrous dichloromethane. The orange tritylium ion solution changed to colorless in a few seconds. The mixture was stirred for additional 10 min, and the solvent was removed by vacuum distillation. The residue were purified by silica column chromatography (pure hexane) to obtained pure triphenylmethane (Tr-H) as white powders (218 mg, 81% yield, Supplementary Figure 16). Hydride-transfer reaction between Tr^+ (247 mg, 0.748 mmol) and N-DMBI (200 mg, 0.748 mmol) was performed using similar procedure to obtained Tr-H (159 mg, 87% yield). ^1H NMR (CD_2Cl_2, 400 MHz, ppm): δ 7.20 (m, 6H), 7.12 (m, 3H), 7.04 (m, 6H), 5.46 (s, 1H). ^{13}C NMR (CD_2Cl_2, 101 MHz, ppm): δ 144.0, 129.3, 128.3, 126.3, 56.8. Both TAM and N-DMBI can transfer hydride to strong electrophile in high yield.

Supplementary Note 5 | Hydride-transfer reaction rates. The apparent rate constants (k_{obs}) for pseudo first order reaction are obtained by linear fitting of time-dependent absorbance logarithm (Supplementary Figure 17)[2]. From Beer–Lambert law, the absorbance is the product of molar absorption coefficient (ε), sample path length (l), and concentration (c):

$$A = \epsilon \cdot l \cdot c$$

For pseudo first order reaction, the concentration variation rate is the production of apparent rate constants (k_{obs}) and c:

$$-\frac{dc}{dt} = k_{obs} \cdot c$$

The above differential equation have the solution of this form, where c_0 is the initial concentration, and t_0 is the time when reaction starts:

$$\ln(c - c_0) = -k_{obs}(t - t_0)$$

Therefore, the logarithm of absorbance has a simple linear relationship with time, the opposite of slope is k_{obs} and b represents for the intercept.

$$\ln(\mathrm{A}) = -k_{obs} \cdot t + [k_{obs} \cdot t_0 + \ln(c_0) + \ln(\epsilon) + \ln(l)] = -k_{obs} \cdot t + b$$

The apparent rate constants are calculated to be 2.61×10^{-4} s^{-1} for TAM and 7.40×10^{-2} s^{-1} for N-DMBI. These results demonstrate N-DMBI has a 284 times faster hydride-transfer reaction rate than TAM.

Supplementary Note 6 | Hydride kinetic nucleophilicity. TAM shows higher activation Gibbs free energy (12~16 kcal/mol higher) in hydride-transfer reactions with each electrophiles than N-DMBI, and TAM will show much slower reaction rates in these hydride-transfer reaction than N-DMBI (Supplementary Figure 18). Kinetic hydride nucleophilicity (N) are calculated from transition state theory-based equation and Mayr equation[3-6]:

$$k_{298K} = k_0 \frac{k_B T}{h} \exp[\frac{-\Delta G^{\ddagger}_{298K}}{RT}]$$

$$\log_{10}(k_{298K}) = s_N(N + E)$$

Where k_{298} is rate constant, k_0 is a factor to guarantee units of M^{-1} s^{-1} for the dimensions of k_{298}, k_B, h, R are the Boltzmann, Planck and gas constants, T is absolute temperature, $\Delta G^{\ddagger}_{298K}$ is the activation Gibbs free energy, s_N and N are is sensitivity parameter and kinetic nucleophilicity of nucleophile, and E is electrophilicity of electrophile. The kinetic hydride nucleophilicity (N) of TAM and N-DMBI are calculated to be 5.79 for TAM (s_N = 0.68) and 17.80 for N-DMBI (s_N = 0.87). This large kinetic hydride nucleophilicity of 17.8 suggest that strong nucleophile N-DMBI would have fast reaction rates with typical electrophiles, while TAM with its smaller nucleophilicity of 5.79 will have slow reaction rates with weak electrophiles in room temperature. These results also indicating that TAM needs thermal activation to react with weak electrophiles.

Supplementary Note 7 | Thermoleg thickness dependent thermoelectric performance[7-10]. Power conversion efficiency maximum (η_{max}) of thermoelectric generators is determined by working temperatures and ZT of materials, while power output maximum (P_{max}) is determined by Seebeck coefficients and internal resistances (R) of materials and working temperatures:

$$\eta_{max} = \frac{T_h - T_c}{T_h} \cdot \frac{\sqrt{1 + ZT} - 1}{\sqrt{1 + ZT} + \frac{T_c}{T_h}} \quad (1)$$

$$P_{max} = \frac{N \cdot {S_{tot}}^2 \cdot (T_h - T_c)^2}{4R} \quad (2)$$

where T_h and T_c are temperatures of the hot side and cold side of thermolegs, N is the amount of p-n thermoleg couples, and S_{tot} is the sum of absolute value of Seebeck coefficients of p-type and n-type materials[11].

Single-thermoleg thermoelectric generator model was used to elaborate the thickness-dependent vertical device performance (Supplementary Figure 25). The device works between two heat source: high temperature solid heat source (T_{hot}) and low temperature air heat source T_{cold}. Since thermal resistance of air is too large, so heat sink is indispensable to ensure the temperature gradient is mainly located in thermoleg. The contact thermal resistances and thermal resistances from high temperature heat source are ignored.

Fourier's law of heat conduction pinpoints the heat transfer in thermoelectric generator:

$$\Delta Q = -\kappa \cdot A \cdot \nabla T \cdot \Delta t \quad (3)$$

where ΔQ is the heat transfer during time Δt, ∇T is temperature gradient, κ is thermos conductivity, A is heat transfer area. In thermoelectric generator with power conversion efficiency (PCE) less than 5%, the converted heat could be ignored. Thus, the heat flux cross thermoleg and heat sink is given by Supplementary Equation 4:

$$\Phi = \frac{\Delta Q}{\Delta t} = -\kappa \cdot A \cdot \frac{T_{hot} - T_i}{h} = -\kappa_{sink} \cdot A_{sink} \cdot \frac{T_i - T_{cold}}{h_{sink}} \quad (4)$$

where Φ is heat flux, h is thickness of thermoleg, h_{sink} is thickness of heat sink, κ_{sink} is thermal conductivity of heat sink, A_{sink} is effective heat transfer area of heat sink, T_i is the temperature of interface between thermoleg and heat sink. Thus, the effective temperature difference (ΔT_{active}) in thermoleg is given by Supplementary Equation 5 as a Hill-type equation:

$$\Delta T_{active} = T_{hot} - T_i = \frac{\frac{\kappa_{sink} \cdot A_{sink}}{h_{sink}}}{\frac{\kappa \cdot A}{h} + \frac{\kappa_{sink} \cdot A_{sink}}{h_{sink}}} \cdot (T_{hot} - T_{cold}) \quad (5)$$

As maximum PCE (η_{max}) is effective temperature difference dependent:

$$\eta_{max} = \frac{\Delta T_{active}}{T_{hot}} \cdot \frac{\sqrt{1 + Z\overline{T}} - 1}{\sqrt{1 + Z\overline{T}} + \frac{T_{cold}}{T_{hot}}} \quad (6)$$

$$P_{max} = \frac{S^2 \cdot (\Delta T_{active})^2}{4R} \quad (7)$$

Thus, maximum PCE is thickness dependent:

$$\eta_{max} = \frac{\frac{\kappa_{sink} \cdot A_{sink}}{h_{sink}}}{\frac{\kappa \cdot A}{h} + \frac{\kappa_{sink} \cdot A_{sink}}{h_{sink}}} \cdot \frac{T_{hot} - T_{cold}}{T_{hot}} \cdot \frac{\sqrt{1 + Z\overline{T}} - 1}{\sqrt{1 + Z\overline{T}} + \frac{T_{cold}}{T_{hot}}} \quad (8)$$

We performed a simple numerical modeling to visualize thickness-dependent maximum PCE (Supplementary Figure 26). In this modeling, we chose T_{cold} = 298 K, T_{hot} = 398 K. Heat sink materials is chosen to be widely used aluminum with thermal conductivity of 57 W m^{-1} K^{-1}, thickness of heat sink is 0.5 mm to 2 mm to ensure its mechanical strength and effectively cooling. The effective heat transfer area of heat sink is chosen to be four times of heat transfer area of thermoleg as $A_{sink} = 4A$. Thermoleg materials is chosen to be PEDOT:PSS (DMSO-mixed) with electrical conductivity of 880 S cm^{-1}, Seebeck coefficient of 73 μV K^{-1}, power factor of 469 μW m^{-1} K^{-2}, thermal conductivity of 0.33 W m^{-1} K^{-1}, ZT of 0.42 at 297 K, which is the highest in organic thermoelectric materials. Power factor and ZT is assumed remain nearly no changed during the modeling temperature. Thickness-dependent maximum PCE is plotted in Supplementary Figure 26.

Compare to thin thermoleg (10 nm), vertical thermoelectric generator with thick thermolegs (1 ~ 10 μm) shows largely enhanced maximum PCE.

The maximum output power is also effective temperature difference dependent:

$$P_{max} = \frac{S^2 \cdot (\Delta T_{active})^2}{4R} \tag{S9}$$

where R is internal electrical resistance of thermoleg, S is Seebeck coefficient. Internal electrical resistance is proportional to thermoleg thickness:

$$R = \frac{1}{\sigma} \cdot \frac{h}{A} \tag{10}$$

where σ is electrical conductivity of thermoleg. According to the definition of power factor (PF):

$$PF = \sigma \cdot S^2 \tag{11}$$

Thus, the maximum output power per unit area is given by Supplementary Equation 12:

$$\frac{P_{max}}{A} = \frac{1}{4h} \cdot PF \cdot (\Delta T_{active})^2 \tag{12}$$

According to Supplementary Equation 5 and Supplementary Equation 12, the maximum output power per unit area is also thickness-dependent:

$$\frac{P_{max}}{A} = \frac{1}{4h} \cdot PF \cdot \left(\frac{\frac{\kappa_{sink} \cdot A_{sink}}{h_{sink}}}{\frac{\kappa \cdot A}{h} + \frac{\kappa_{sink} \cdot A_{sink}}{h_{sink}}} \right)^2 \cdot (T_{hot} - T_{cold})^2 \tag{13}$$

Numerical modeling of thickness-dependent maximum output power is plotted in Supplementary Figure12c. Compare to thin thermoleg (10 nm), vertical thermoelectric generator with thick thermolegs (1 ~ 10 μm) shows much enhanced maximum output power.

Single-thermoleg thermoelectric generator model was used to elaborate the thickness-dependent lateral device performance (Supplementary Figure 27). Similar to vertical thermoelectric generator, the device also works between two heat source T_{hot} and T_{cold}. The lateral device could be fabricated as large as temperature difference loss is negligible. However, lateral thermoelectric generator is also difficult to be "free standing" and a substrate is indeed. Thus, heat transfer through substrate would cause non-ignorable heat loss. In lateral thermoelectric generator with power conversion efficiency (PCE) less than 5%, the converted heat could also be ignored. The contact thermal resistances and thermal resistances of electrode can be also ignored. Thus, the total heat transfer (Q_{total}) through the whole single-thermoleg device is given by Supplementary Equation 14:

$$Q_{total} = Q_{active} + Q_{loss} + W_{electrical} \approx Q_{active} + Q_{loss} \tag{14}$$

Where Q_{active} is the heat that transfering through thermoleg, Q_{loss} is the heat that transfering through substrate. From Fourier's law of heat conduction, Q_{active} and Q_{loss} are given by Supplementary Equation 15:

$$\begin{cases} Q_{active} = -\kappa \cdot w \cdot h \cdot \nabla T \cdot \Delta t \\ Q_{loss} = -\kappa_{sub} \cdot w \cdot h_{sub} \cdot \nabla T \cdot \Delta t \end{cases} \tag{15}$$

From Supplementary Equation 13, heat transfer efficiency is given by Supplementary Equation 16:

$$\frac{Q_{active}}{Q_{total}} = \frac{Q_{active}}{Q_{active} + Q_{loss}} = \frac{\kappa \cdot h}{\kappa \cdot h + \kappa_{sub} \cdot h_{sub}} \tag{16}$$

Thus, the maximum PCE in lateral device is thickness dependent:

$$\eta_{max} = \frac{Q_{active}}{Q_{total}} \cdot \frac{\Delta T_{active}}{T_{hot}} \cdot \frac{\sqrt{1 + Z\bar{\bar{T}}} - 1}{\sqrt{1 + Z\bar{\bar{T}}} + T_{cold}/T_{hot}} \tag{17}$$

$$\eta_{max} = \frac{\kappa \cdot h}{\kappa \cdot h + \kappa_{sub} \cdot h_{sub}} \cdot \frac{\Delta T_{active}}{T_{hot}} \cdot \frac{\sqrt{1 + Z\bar{\bar{T}}} - 1}{\sqrt{1 + Z\bar{\bar{T}}} + T_{cold}/T_{hot}} \tag{18}$$

The internal electrical resistance in lateral device is inversely proportional to thermoleg thickness:

$$R = \frac{1}{\sigma} \cdot \frac{l}{w \cdot h} \tag{19}$$

Considering Supplementary Equation 9 and Supplementary Equation 11, the maximum output power is given by Supplementary Equation 20:

$$P_{max} = \frac{w}{4l} \cdot PF \cdot (\Delta T_{active})^2 \cdot h \propto h \tag{20}$$

Thus, the maximum output power is proportional to thermoleg thickness. We also performed simple numerical modeling to visualize thickness-dependent maximum PCE and maximum output power in the lateral device (Supplementary Figure 28). In this modeling, T_{cold} = 298 K and T_{hot} = 398 K, thermoleg materials of PEDOT:PSS (DMSO-mixed) with electrical conductivity of 880 S cm^{-1}, Seebeck coefficient of 73 μV K^{-1}, power factor of 469 μW m^{-1} K^{-2}, thermal conductivity of 0.33 W m^{-1} K^{-1}, *ZT* of 0.42 at 297 K were also used. Power factor and *ZT* is assumed remain nearly no changed during the modeling temperature. A square thermoleg with $l = w$ were also used to simplified calculations. We chose polyimide (PI) and polyethylene terephthalate (PET) with thermal conductivity of 0.20 W m^{-1} K^{-1} and thickness of 5 ~ 20 μm as the substrate materials. To ensure the mechanical strength, the thickness of these flexible substrate could not be less than 5 μm. Thickness-dependent maximum PCE and maximum output power are plotted in Supplementary Figure 28. Compare to thin thermoleg (10 nm), lateral thermoelectric generator with thick thermolegs (1 ~ 10 μm) shows greatly enhanced maximum PCE and output power.

Supplementary Note 8 | Stability of electrical conductivity. Time-dependent electrical conductivity measurement shows that TAM-doped FBDPPV thin films are unstable in air, while the thick film is stable in air (Supplementary Figure 29). This indicates that TAM-doped FBDPPV thick film can form self-encapsulating to prevent bulk oxidative de-doping under room temperature. Long-term continuous annealing measurement shows that TAM doped FBDPPV thick film is also stable at high temperature under nitrogen. This would imply that TAM doping is stable and irreversible. Notice that TAM-doped FBDPPV thick film is unstable during heating in air, suggesting that the self-encapsulating is invalid at high temperature. CYTOP was employed to encapsulate TAM-doped FBDPPV thick film. However, the CYTOP encapsulate layer can only slow down but cannot prevent the oxidative de-doping. Thus, more effective encapsulation methods need to be developed in the future studies.

Supplementary Note 9 | Temperature dependent thermoelectric properties. Both TAM and N-DMBI doped FBDPPV shows slightly higher electrical conductivity at higher temperature (Supplementary Figure 33a). This thermally activated conduction could be described by the nearest-neighbor hopping (NNH) mechanism (Supplementary Figure 33b)[12-14]. In this NNH conduction,

both TAM and N-DMBI doped FBDPPV show weak temperature dependent electrical conductivity with small activation energy of 23.6 meV and 33.4 meV, respectively (Supplementary Figure33c). Notice that TAM doped FBDPPV shows slightly weaker temperature dependence of electrical conductivity than N-DMBI doped FBDPPV. This indicates that TAM doped FBDPPV would have lower molecular disorder in solid state[15-17], which is consistent with the GIWAXS analysis.

Both TAM and N-DMBI doped FBDPPV shows slightly higher Seebeck coefficient at higher temperature (Supplementary Figure 34a). Notice that TAM doped FBDPPV shows slightly weaker temperature dependence of Seebeck coefficient ($\partial S/\partial(1/T)$ = 14.9 meV) than N-DMBI doped FBDPPV ($\partial S/\partial(1/T)$ = 27.1 meV). This indicates that TAM doped FBDPPV could have lower molecular disorder in solid state[15-17], which is consistent with GIWAXS analysis.

Supplementary Note 10 | TD-DFT calculations on absorption spectra. The geometry configuration of FBDPPV trimer, (FBDPPV trimer)$^{-}$ anion, and (FBDPPV trimer)$^{2-}$ dianion were firstly optimized, then the first 50 vertical excitation energy (ΔE_{ve}) and their corresponding oscillator strength of each compound were calculated (Supplementary Table 4-6). Finally, the absorption spectra were generated using Gaussian peak fitting with half-width at half height of 0.10 eV for each peak. Geometry optimizations and TD-DFT calculations were under B3LYP/6-31G(d) level, long alkyl chains were replaced with methyl to simplify the calculation.

TD-DFT calculations[16] reveal that in TAM doped FBDPPV (140 °C) and N-DMBI doped FBDPPV (room temperature), the absorption bands (900 to 1400 nm, Band III; 1400 to 2000 nm, Band IV) at long wavelength are distinct from the absorption of intrinsic FBDPPV (400 to 650 nm, Band I; 650 to 900 nm, Band II), which can be attributed to polaronic absorption of negatively charged FBDPPV polymer. Furthermore, Band III might be attributed to the absorption of polaron anions, and Band IV might be attributed to the absorption of bipolaron dianions.

Supplementary Note 11 | Coherence length and paracrystalline disorder in GIWAXS analysis. Coherence length (L_c) is calculated from breadth (Δ_q) of a diffraction peak (Supplementary Figure 42-43)[18, 19]:

$$L_c = 0.89 \times \frac{2\pi}{\Delta_q}$$

And paracrystalline disorder is calculated from the center position (q_0) and breadth (Δ_q)[S18, S19]:

$$g = \sqrt{\frac{\Delta_q}{2\pi q_0}}$$

Supplementary Methods

General procedures and experimental details. All chemicals and solvents are of reagent grade unless otherwise indicated. All air and water sensitive reactions were performed under nitrogen atmosphere.

^1H and ^{13}C NMR spectra were recorded on Bruker ARX-400 (400 MHz). All chemical shifts were reported in parts per million (ppm). ^1H NMR chemical shifts were referenced to TMS (0 ppm), and ^{13}C NMR chemical shifts were referenced to $CDCl_3$ (77.0 ppm). Molecular weights were determined by gel permeation chromatography (GPC) performed on Polymer Laboratories PL-GPC220 at 150 ºC using 1,2,4-tricholorobenzene (TCB) as eluent. Absorption spectra were recorded on PerkinElmer Lambda 750 UV-vis spectrometer.

Ultraviolet photoemission spectroscopy (UPS) and X-ray photoelectron spectroscopy (XPS) were conducted on a Kratos AXIS Ultra-DLD Photoelectron Spectrometer under an ultrahigh vacuum of about 3×10^{-9} Torr with an unfiltered He I gas discharge lamp source (21.22 eV) and a monochromatic Al Kα source (1486.6 eV) as the excitation source, respectively. For UPS measurements, the samples were biased at -9 V to observe the low-energy secondary electron cutoff. The instrumental energy resolution for UPS and XPS were 0.1 eV and 0.5 eV, respectively. Before measurements, all the samples were spin-coated on 1 cm × 1 cm native oxide silicon substrates (detailed process was same as for OFET devices) in a N_2 glove box and transferred through a transport system without air exposure in to the spectrometer analysis chamber.

The 2D-GIWAXS data were obtained at beamline BL14B1 of the Shanghai Synchrotron Radiation Facility (SSRF) at a wavelength of 1.236 Å. BL14B1 is a beamline based on bending magnet and a Si (111) double crystal monochromator was employed to monochromatize the beam. The size of the focus spot is about 0.5 mm and the end station is equipped with a Huber 5021 diffractometer. NaI scintillation detector was used for data collection.

Solution doping and film fabrication. FBDPPV, N2200, TAM and N-DMBI were dissolved in trichloroethylene (TCE) or 1,2-dichlorobenzene (ODCB). Aliquots of TAM and polymer solutions were mixed at room temperature and stirred for 10 min. Films were deposited on the treated substrates by spin-coating or drop-casting the mixed solutions, and annealed at 140 °C for 8 h. Film thickness of each samples were measured by atomic force microscope (< 100 nm) and profile meter (100 nm ~ 10 μm).

FET devices fabrications and testing. FET measurements are similar to our previous studies (Jian Pei *et al*, *Adv. Mater.* 2018, *30*, 1802850.). TG/BC FET devices were fabricated using n^{++}-Si/SiO_2 (300 nm) substrates. The gold source and drain bottom electrodes (with Ti as the adhesion layer) were patterned by photolithography on the SiO_2 surface. The substrates were subjected to cleaning using ultrasonication in acetone, detergent, deionized water (twice), and isopropyl alcohol. The cleaned substrates were dried by nitrogen. A thin film of the polymer was deposited on the treated substrates by spin-coating the polymer solution or polymer-dopant mixture solution (3 mg mL^{-1} in TCE) at 2000 rpm for 60 s in glovebox, optionally followed by thermal annealing. After the deposition of the polymer thin film, a CYTOP solution (CTL809M:CT-solv180 = 3:1) was spin-coated onto the semiconducting layer at 2000 rpm for 60 s, resulting in a 500 nm thick dielectric layer. The CYTOP layer was then annealed at 100 °C for 60 min in the glovebox. Gate electrodes comprising a layer of Al (70 nm) were then evaporated through a shadow mask onto the dielectric layer under high vacuum (10^{-4} Pa). The OFET devices have a channel length of 20 μm and a channel

width of 400 μm. The evaluations of the FETs were carried out under ambient conditions (25 °C, R_H = 50-60%) on a probe stage using a Keithley 4200SCS as parameter analyzer. The carrier mobility (μ) was calculated from the data in the saturated regime according to the equation:

$$I_{SD} = \frac{W}{2L} C_i \mu (V_G - V_T)^2$$

Where I_{SD} is the drain current in the saturated regime. W and L are, respectively, the semiconductor channel width and length, C_i (C_i = 3.7 nF) is the capacitance per unit area of the gate dielectric layer, and V_G and V_T are the gate voltage and threshold voltages. $V_G - V_T$ of the device was determined from the relationship between the square root of I_{SD} and V_G at the saturated regime.

Thermoelectric properties measurements. Thermoelectric properties measurements are similar to our previous studies (Jian Pei *et al*, *J. Am. Chem. Soc.* **2015**, 137, 6979- 6982; Jian Pei *et al*, *Angew. Chem. Int. Ed.* **2019**, *58*, 11390-11394.). All devices were fabricated using glass substrates. The gold electrodes were pre-patterned by photolithography on the surface with a channel length of 100 μm and a channel width of 500 μm for conductivity measurements and a channel length of 5 mm and a channel width of 500 μm for Seebeck coefficient measurements (Supplementary Figure 54). The substrates were subjected to cleaning using the same procedures as above. Thin films were deposited on the treated substrates by spin-casting or drop-casting and annealed at 140 °C for 8 h. 4-Point conductivity measurements were conducted in an N_2 glovebox with Keithley 4200SCS. The Seebeck coefficient measurements were performed in vacuum. Electrical conductivity was measured with a co-linear four-point-probe bar geometry (D = 50 μm). Seebeck measurement contacts consisted of 0.1 mm × 0.5 mm electrical contact bars, 5 mm apart. The Seebeck coefficient is calculated by

$$S = \frac{V_{therm}}{\Delta T}$$

Where V_{therm} is the thermal voltage obtained between the two ends of the device subject to a temperature gradient ΔT. The V_{therm} was measured with Keithley 4200 SCS, and the temperature difference was introduced by Joule heat (heater) and liquid nitrogen cooling system. The temperature coefficient of resistance (TCR) of the temperature sensing wires was calculated from the slope of the measured resistance versus temperature. The resistance of the metal wires is linear correlation with the temperature. TCR was found to be 0.307 Ω K^{-1} with R^2 = 0.9999. By monitoring the resistance evolution of the temperature sensing electrodes, the accurate temperature of the contact pads was figured out by $T_h = T_{r.t.} + (R_h - R_{r.t.})$/TCR and $T_c = T_{r.t.} + (R_c - R_{r.t.})$/TCR. The temperature difference was then known as the difference in temperature between the hot and the cold ends $\Delta T = T_h - T_c$. The device architecture for electrical conductivity and Seebeck coefficient measurements is shown as Supplementary Figure 54.

Supplementary References

1. Brown T. L. The electronic properties of alkyl groups. I. Dispersion and induction forces. *J. Am. Chem. Soc.* **81**, 3229-3231 (1959).
2. Horn M., et al. Towards a comprehensive hydride donor ability scale. *Chem. Eur. J.* **19**, 249-263 (2013).
3. Richter D., Mayr H. Hydride-donor abilities of 1,4-dihydropyridines: A comparison with pi nucleophiles and borohydride anions. *Angew. Chem. Int. Ed.* **48**, 1958-1961 (2009).
4. Alherz A., et al. Predicting hydride donor strength via quantum chemical calculations of hydride transfer activation free energy. *J. Phys. Chem. B.* **122**, 1278-1288 (2018).
5. Horn M., Mayr H. Electrophilicities of acceptor-substituted tritylium ions. *Eur. J. Org. Chem.* **2011**, 6470-6475 (2011).
6. Zhuo L.-G., et al. A frontier molecular orbital theory approach to understanding the mayr equation and to quantifying nucleophilicity and electrophilicity by using homo and lumo energies. *Asian J. Org. Chem.* **1**, 336-345 (2012).
7. Glatz W., et al. Optimization and fabrication of thick flexible polymer based micro thermoelectric generator. *Sens. Actuator A Phys.* **132**, 337-345 (2006).
8. Huesgen T., et al. Design and fabrication of MEMS thermoelectric generators with high temperature efficiency. *Sens. Actuator A Phys.* **145-146**, 423-429 (2008).
9. Jang B., et al. Optimal design for micro-thermoelectric generators using finite element analysis. *Microelectron. Eng.* **88**, 775-778 (2011).
10. Cheng F., et al. A thermoelectric generator for scavenging gas-heat: From module optimization to prototype test. *Energy* **121**, 545-560 (2017).
11. Kroon R., et al. Thermoelectric plastics: from design to synthesis, processing and structure-property relationships. *Chem. Soc. Rev.* **45**, 6147-6164 (2016).
12. Thorpe M. F., et al. Electronic density of states of amorphous Si and Ge. *Phys. Rev. Lett.* **27**, 1581-1584 (1971).
13. Yildiz A., et al. Crossover from nearest-neighbor hopping conduction to Efros-Shklovskii variable-range hopping conduction in hydrogenated amorphous silicon films. *Japan. J. Appl. Phys.* **48**, 111203, (2009).
14. Yang C. Y., et al. Enhancing the n-type conductivity and thermoelectric performance of donor-acceptor copolymers through donor engineering. *Adv. Mater.* **30**, 1802850 (2018)
15. Russ B., et al. Organic thermoelectric materials for energy harvesting and temperature control. *Nat. Rev. Mater.* **1**, 1-14 (2016).
16. Wang S. H., et al. Thermoelectric properties of solution-processed n-doped ladder-type conducting polymers. *Adv. Mater.* **28**, 10764-10771 (2016).
17. Liu, J., et al., Enhancing molecular n-type doping of donor-acceptor copolymers by tailoring side chains. *Adv. Mater.* **30**, 1704630 (2018).
18. Rivnay J., et al. Quantitative analysis of lattice disorder and crystallite size in organic semiconductor thin films. *Phys. Rev. B* **84**, 045203 (2011).
19. Noriega R., et al. A general relationship between disorder, aggregation and charge transport in conjugated polymers. *Nat. Mater.* **12**, 1037-1043 (2013).
20. Sondergaard R. R., et al. Practical evaluation of organic polymer thermoelectrics by large-area R2R processing on flexible substrates. *Energy Sci. Eng.* **1**, 81-88 (2013).
21. Du Y., et al. Thermoelectric Fabrics: Toward Power Generating Clothing. *Sci. Rep.* 5, 6411 (**2015**).

22. Wei Q., et al. Polymer thermoelectric modules screen-printed on paper. *Rsc. Adv.* **4**, 28802-28806 (2014).
23. Kim N., et al. Elastic conducting polymer composites in thermoelectric modules. *Nat. Commun.* **11**, 1424 (**2020**).
24. Jiao F., et al. Inkjet-printed flexible organic thin-film thermoelectric devices based on p- and n-type poly(metal 1,1,2,2-ethenetetrathiolate)s/polymer composites through ball-milling. *Philos. T. R. Soc. A* 372, 20130008 (**2014**).
