# Supplementary material for: A thermally activated and highly miscible dopant for n-type organic thermoelectrics
Source: Nat Commun. 2020 Jul 3;11:3292. doi: 10.1038/s41467-020-17063-1 (PMC7335177; doi:10.1038/s41467-020-17063-1)
Supplement: Supplementary file 3 — Description of Additional Supplementary Files [file 41467_2020_17063_MOESM3_ESM.pdf]

Title: Supplementary Movie 1
Description: Testing of all-polymer thermoelectric generator.
